# Association between emotion recognition and cognitive functioning in multiple sclerosis: a multilevel correlational meta-analysis

**DOI:** 10.1038/s41598-026-52969-8

**Published:** 2026-05-29

**Authors:** Béatrice Degraeve, Audrey Henry, Bruno Lenne

**Affiliations:** 1 Neurethic Lab / FLSH - ETHICS EA7446, Université Catholique de Lille, F-59000 Lille, France; 2 Rehabilitation Department, Groupement des Hôpitaux de l’Institut Catholique de Lille (GHICL), Hôpital Saint-Philibert, Lomme, France; 3 Université de Reims Champagne-Ardenne, Laboratoire C2S (ER 6291), Reims, France; 4 Pôle Universitaire de Psychiatrie, EPSM de Reims, Reims, France; 5 Neurology Department, Groupement des hôpitaux de l’institut catholique de Lille (GHICL), Lille, France

**Keywords:** Multiple sclerosis, Emotion recognition, Meta-analysis, Cognitive impairment, Social cognition, Neuroscience, Psychology, Psychology

## Abstract

Multiple sclerosis (MS) is a chronic neuroinflammatory and neurodegenerative disease marked by both cognitive and socio-cognitive impairments, including difficulties in recognizing emotions. While deficits in processing speed, executive functions, and episodic memory are well known, growing evidence also points to impairments in emotion-recognition, particularly for negative emotions such as fear, sadness, or disgust. However, the extent to which these socio-cognitive difficulties are linked to broader cognitive dysfunction remains unclear, as individual studies report inconsistent findings. This meta-analysis synthesized data from 23 independent studies (*k* = 774 correlations) using multilevel random-effects models to quantify the association between emotion-recognition and cognitive functioning in MS. Overall, results revealed small to moderate associations between emotion-recognition and cognitive performance, with stronger effects for negative emotions and for total emotion scores. Processing speed and executive functions exhibited broad associations, while episodic memory effects were more emotion-specific. At the subdomain level, working memory and verbal fluency emerged as key correlates. However, substantial heterogeneity across studies should be noted. Additional meta-regressions indicated that higher disability and anxiety were associated with stronger emotion–cognition links, whereas longer disease duration and depression predicted weaker associations. These findings indicate that emotion-recognition deficits in MS seem partially connected to neurocognitive dysfunctions.

**Trial registration**: The preregistered review protocol can be accessed at 10.17605/OSF.IO/3VNA4.

**Supplementary Information:**

The online version contains supplementary material available at 10.1038/s41598-026-52969-8.

## Introduction

### Background

Multiple sclerosis (MS) is a chronic disease of the central nervous system, marked by inflammatory activity and progressive neurodegeneration, resulting in multifocal demyelination and axonal loss^1,2^. MS typically manifests during early adulthood and represents the most common cause of non-traumatic neurological disability in this population^3^. The disease is characterized by a broad and unpredictable range of symptoms, including motor, visual, neuropsychiatric symptoms, and cognitive decline^4^ which can severely affect autonomy and daily functioning^5^. Cognitive deficits are reported in up to 70% of individuals with MS (pwMS), regardless of disease stages or clinical subtypes^6^, and contribute substantially to functional impairment^4,5^. The most frequently affected domains include information processing speed (IPS)^6,7^, episodic memory, and executive functioning^6,8^. Beyond cognitive dysfunction, growing evidence points to impairments in social cognition in MS^9,10^, including in early-stage disease and in patients with clinical isolated syndromes (CIS)^11–14^.

Social cognition is a multi-component construct referring to a set of different processes that allow individuals to perceive, interpret, and respond appropriately to social cues^15,16^. Core components of social cognition include social perception (e.g., emotion recognition), mental state decoding (e.g., theory of mind, ToM), empathy, and social behavior^16^. Meta-analyses have confirmed the presence of deficits in both ToM and emotion recognition among pwMS^9,17–19^. Although studies generally agree that emotion recognition deficits in pwMS mainly concern negative emotions, the mechanisms underlying these difficulties remain debated. Functional MRI studies have highlighted altered activation in neural circuits supporting socio-emotional processing^14,20,21^, while others pointed to a potential disconnection between cortical and subcortical regions caused by demyelination and axonal damage (see^22^ for a review).

Beyond neuroanatomical accounts, an ongoing debate in the literature also concerns whether these impairments reflect broader cognitive dysfunction or constitute a distinct clinical feature in MS (for a review, see^23^. This question has been addressed so far through behavioral evidence, by examining whether social cognition impairments in MS can be accounted for by co-occurring deficits in general cognitive domains, based on associations observed in neuropsychological performance. While some studies reported moderate correlations between social and non-social cognitive domains, suggesting partial overlap^10,24^, others have failed to observe such links and proposed that emotion recognition deficits may arise independently of global neurocognitive impairment^25,26^. The relationships with clinical data (disease duration, MS subtypes, disability level, fatigue) also remain unclear. Given that these mixed findings may stem in part from limited statistical power across individual studies or methodological heterogeneity, a meta-analysis can be helpful to increase statistical power and clarify these conclusions. Understanding the link between specific domains of cognition and emotion recognition in people with MS (pwMS) would be useful for identifying cognitive targets and developing intervention programs.

### Objectives

The relationship between emotion recognition and cognitive functioning in MS has been examined in numerous studies, yet findings remain inconsistent. To date, no meta-analytic review has systematically quantified the strength of this association across the available literature. Moreover, it is still unclear under which clinical or demographic conditions this association is more likely to emerge.

The present meta-analysis thus aimed to address this gap by estimating the overall magnitude of the association between emotion recognition performance and cognitive functioning in pwMS. Specifically, we assessed correlations across seven emotion scores of interests (a total score and six basic emotion subscores) and three cognitive domains (information processing speed, executive functions, and episodic memory). Given prior evidence suggesting that negative emotions are more difficult to recognize than positive ones^27^ and that emotion recognition deficits in pwMS appear to be predominantly restricted to negative emotions (particularly anger, fear, and sadness^9,17^, we considered it important to account for emotion type in our analyses. Disaggregating the total emotion recognition score into basic emotion sub-scores allowed us to examine whether the strength of the association with cognitive functioning differs depending on emotional valence or specific emotional categories.

In addition to emotion type, it is also important to consider cognitive domains and identify those most strongly associated with emotion recognition performance. Indeed, the cognitive profile of pwMS is highly heterogeneous^28^, and not all domains are equally affected across individuals^29,30^. Although previous studies have reported positive associations between emotion recognition and various cognitive domains — including executive functioning^10,12,31,32^, episodic memory and IPS^33,34^— the strength and replicability of these associations remain variable across the literature. This variability underscores the importance of analyzing each domain separately to identify which aspects of cognition are most strongly related to social cognitive performance.

Finally, to clarify *for whom* such associations might be relevant, we examined whether the strength of the correlation between emotion recognition and cognitive functioning varies as a function of demographic and clinical characteristics in pwMS. Specifically, we examined potential moderating effects of age, disease duration, disability status (*Expanded Disability Status Scale*, EDSS), depression, anxiety, fatigue, and alexithymia. These variables are known to influence either cognitive performance, emotion recognition, or both, and may help explain between-study variability^6,10,35,36^. In particular, cognitive impairment has been shown to increase with disease duration^29^, and MS phenotypes (CIS, Relapsing-Remitting MS (RRMS), Primary Progressive MS (PPMS), and Secondary Progressive MS (SPMS)^30^ differ in their underlying pathological mechanisms, which may contribute to distinct cognitive and social cognitive profiles^10,29,37^. Therefore, we investigated the moderating role of disease duration and MS subtype (i.e., CIS, RRMS, SPMS, PPMS) in the strength of the observed associations.

In summary, this meta-analysis aims to quantify the overall association between emotion recognition and cognitive impairment in MS, while also assessing the influence of emotion type, cognitive domain, and key clinical or demographic moderators through meta-regression analyses. By clarifying the strength and variability of this relationship, the study contributed to ongoing efforts to determine whether emotion recognition deficits reflect a broader cognitive dysfunction or a distinct symptom dimension. Ultimately, these findings may provide insight into the functional relationship between emotion recognition and cognitive functioning in MS. By identifying the conditions under which these associations are strongest, it may help refine hypotheses about the underlying mechanisms of social cognition impairment and guide future research directions. In clinical settings, the findings may also inform cognitive screening practices by highlighting which cognitive profiles are more likely to present with concomitant social cognitive difficulties.

## Methods

### Eligibility criteria

#### Criteria for inclusion

We conducted a systematic review of the literature to identify all potential eligible published and unpublished studies. Relevant variables were coded in each eligible study, and effect sizes were extracted for quantitative synthesis. Studies were included if they met the following criteria:

1. Studies including (adult) patients diagnosed with MS. Considering that (1) social cognition continues to develop with increased age into adulthood^38^ and (2) that significant neural development and hormonal changes were shown to influence social cognition at puberty^39^, it is not clear whether potential interactions between nonsocial and social cognition are comparable from childhood to adulthood. For this reason, only studies including *adult* MS patients (i.e., aged 18 and over) were included.
2. Studies assessing both emotion recognition and at least one cognitive domain.
3. Studies using standardized and/or recognized measures to assess both emotion recognition and cognitive dysfunction.
4. Studies (or authors) providing correlational measures between emotion recognition and cognition in pwMS (i.e., Pearson, Spearman or point-biserial correlations).
5. Studies published in a peer-reviewed journal in English.
6. Intervention studies were included if authors reported baseline or pre-intervention data.

#### Criteria for exclusion

Studies were excluded at the full-text screening stage if they met the following criteria:

1. Studies not including an (adult) MS patient group. Case studies and studies including pediatric MS patients (i.e., aged < 18) were excluded.
2. The publication is not an empirical original type, such as: research protocols, letters, conference abstracts, reviews, and editorials.
3. Studies with patient samples overlapping with another one with a larger sample size. Indeed, where studies reported data from a subsample of patients from a larger study, only the larger study were included.

### Sample of studies

#### Data sources

**Fig. 1:**
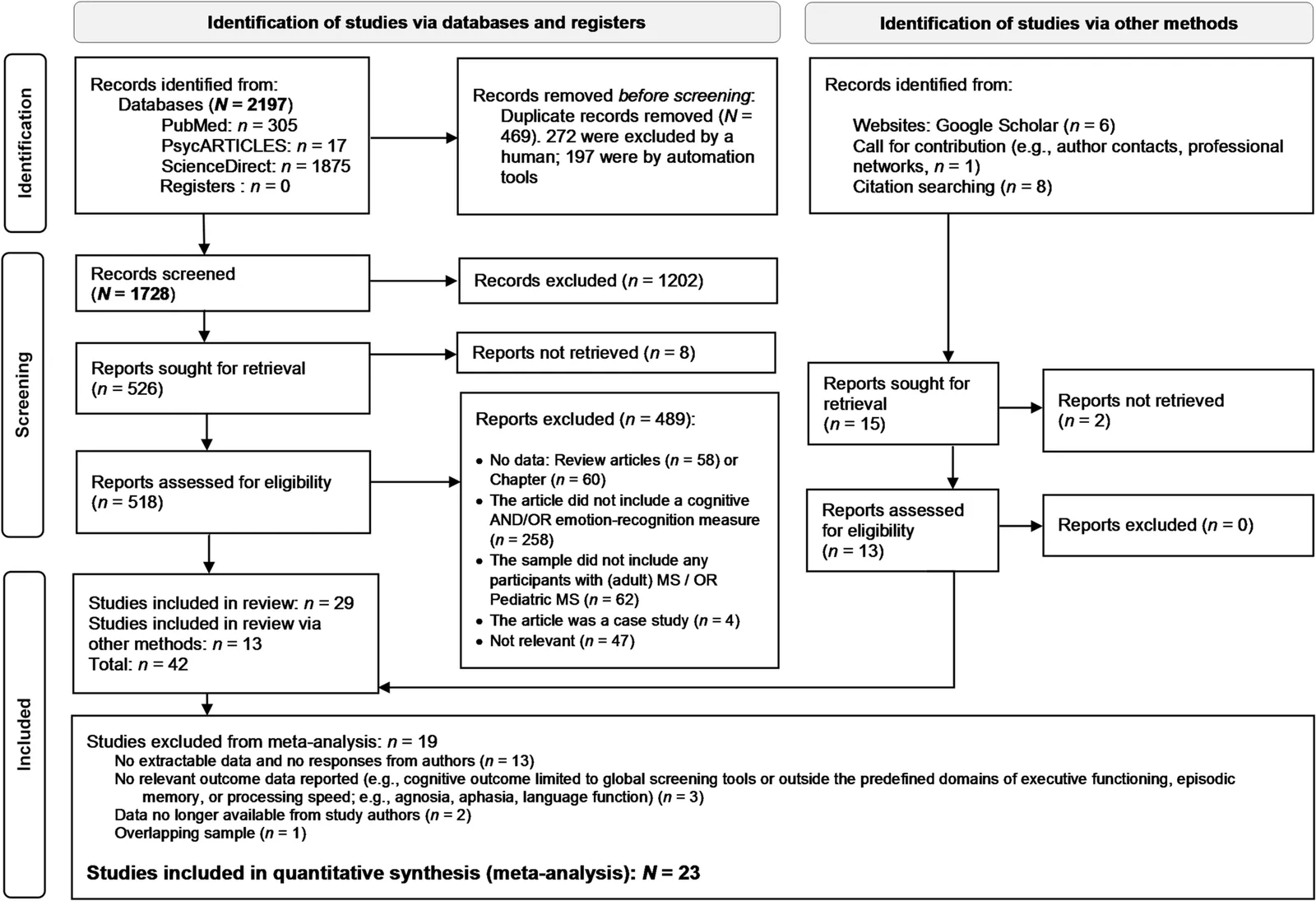
Meta-analysis flow diagram in accordance with PRISMA 2020. Adapted From: Page MJ, McKenzie JE, Bossuyt PM, Boutron I, Hoffmann TC, Mulrow CD, et al. The PRISMA 2020 statement: an updated guideline for reporting systematic reviews. BMJ 2021;372:n71. doi: 10.1136/bmj.n71. http://www.prisma-statement.org/.

A comprehensive search strategy was implemented in an attempt to identify, retrieve, and code the entire population of eligible studies using three electronic databases (PsycARTICLES, PubMed, ScienceDirect) up to 7 January 2024 (see Fig. 1). Additionally, we conducted a search using Google Scholar and reviewed the reference lists of included articles to identify any additional relevant publications that may not be directly indexed in such sources. This search was updated on 2 June 2025 to identify any additional studies published since January 7, 2024.

#### Search strategy and study selection

The literature search was conducted in PubMed, PsycARTICLES, and ScienceDirect using combinations of the following keywords: “multiple sclerosis”, “social cognition”, “emotion”, and “emotion recognition”:

- PubMed: (“multiple sclerosis”) AND (“social cognition” OR “emotion” OR “emotion recognition”).
- PsycARTICLES: (“multiple sclerosis”) AND (“social cognition” OR “emotion” OR “emotion recognition”).
- ScienceDirect: (“multiple sclerosis”) AND (“social cognition” OR “emotion” OR “emotion recognition”).
- Additional sources: Google Scholar and reference lists of included articles were also screened to identify additional relevant studies.

No date restrictions were placed on any searches. There were no restrictions of the age of patients (> 18 years) or phenotype of MS for inclusion.

This search strategy resulted in an initial pool of studies. Study selection was then conducted using a two-stage process, consisting of an initial screening of titles and abstracts followed by a full-text review of potentially eligible articles. At each stage, two authors independently screened records for eligibility according to the predefined inclusion and exclusion criteria. Any uncertainties or disagreements regarding study selection were resolved through discussion with a third author. No dedicated systematic review software was used for the selection process. Instead, records were exported into a standardized data extraction spreadsheet, in which duplicates were identified and removed prior to screening. The study selection process is presented in the PRISMA flow diagram (see Fig. 1).

## Data collection and analysis

A standardized data extraction spreadsheet was used to record data from full-text articles and other sources which meet the eligibility criteria. Specifically, we extracted the following information:

1. Study characteristics (authors, publication year, title, and journal).
2. MS sample characteristics (sample size, age).
3. Clinical characteristics (disease duration, EDSS score, severity of depression, severity of anxiety, fatigue, and alexithymia). Regarding disability, we extracted EDSS scores^40^. EDSS is a widely used clinician-administered assessment that quantifies the severity of disability, increasing from 0 (no disability) to 10 (death due to MS) in increments of 0.5 units.
4. Correlational data provided between emotion recognition performance and cognitive performance in pwMS (i.e., Pearson, Spearman or point-biserial correlations).
5. The type of cognitive and socio-cognitive measures used for each retrieved association (cognitive domain, tasks names).

Where necessary, we contacted study authors for unreported data in order to calculate effect sizes.

## Note on Supplementary material

To optimize the readability and conciseness of the main text, several methodological details have been moved to the Supplementary Material. This includes the list of extracted variables and coding procedures (Section S1), the full classification of cognitive tasks by domain and subdomain (Section S2), the description of the statistical models and estimation procedures (Section S3), and the results of the quality assessment of included studies (Section S4).

## Pre-registration and data availability

This meta-analysis was conducted in accordance with the PRISMA-P guidelines^41,42^. All analyses were pre-registered and followed the protocol published in *BMJ Neurology Open*^43^. To ensure transparency and reproducibility, all extracted data (including the summary Excel file) and supplementary result tables are openly available on the Open Science Framework at 10.17605/OSF.IO/3VNA4. These resources are intended to support replication, verification, and future extensions of the present meta-analysis, providing a ready-to-use framework for researchers who wish to build upon this work.

## Results

### Summary of overall emotion–cognition associations

#### Effects by cognitive domain

To examine emotion–cognition associations, we conducted a multilevel meta-regression on Fisher’s *z*-transformed correlations. Moderator terms for Emotion, Cognitive Domain, and their interaction were entered as fixed effects. For each Emotion—Cognition domain combination, we extracted predicted average effects along with 95% confidence intervals. Significance testing was based on two-tailed *z*-tests with an alpha threshold of 0.05.

Pooled effect sizes based on these marginal estimates revealed positive significant associations in all three domains. All emotions combined, the combined association was small for executive function (*r* = .11, CI_95%_ [0.09, 0.13], *p* < .001), episodic memory (*r* = .17, CI_95%_ [0.13, 0.20], *p* = .007) and IPS (*r* = .16, CI_95%_ [0.13, 0.20], *p* < .001). Overall associations for each emotion–domain pairing were summarized in Table 1.

Across the 24 emotion–domain combinations, most yielded positive associations, with 14 reaching statistical significance. Within the executive function domain, significant effects were observed for Disgust (*r* = .10), Fear (*r* = .13), Sadness (*r* = .09), Surprise (*r* = .10), and Total score (*r* = .22). For episodic memory, only Disgust (*r* = .16), Fear (*r* = .24), and Total Score (*r* = .34) reached significance. Regarding IPS, significant associations emerged for Anger (*r* = .17), Disgust (*r* = .15), Fear (*r* = .19), and Sadness (*r* = .17) and Total Score (*r* = .28).

Fear, Disgust, and Total Score elicited the strongest and most reliable associations with cognitive performance across domains, whereas Happiness and Neutral showed the weakest and least consistent effects.

**Table 1:** Overall associations between emotion recognition and cognitive performance across domains, with heterogeneity indices.

Emotion type	Domain	k	*r*	Lower CI	Upper CI	*p*		$\:\tau\:$ ^2^	$\:\tau\:$	*I* ^2^
Anger	Executive	71	0.09	0.006	0.18	0.03	*	0.02	0.15	43.65
Anger	EM	21	0.14	-0.08	0.35	0.220		0.08	0.28	65.80
Anger	IPS	20	0.16	0.05	0.28	0.006	**	0.02	0.16	36.8
Disgust	Executive	52	0.10	0.04	0.16	0.002	**	0.005	0.07	17.72
Disgust	EM	15	0.16	0.07	0.25	< 0.001	***	0.001	0.001	0.001
Disgust	IPS	16	0.15	0.05	0.25	0.003	**	0.007	0.09	14.73
Fear	Executive	70	0.13	0.06	0.20	< 0.001	**	0.01	0.10	26.48
Fear	EM	21	0.24	0.02	0.44	0.03	*	0.08	0.28	65.66
Fear	IPS	20	0.19	0.06	0.31	0.004	**	0.03	0.17	42.30
Happiness	Executive	70	0.003	-0.08	0.09	0.950		0.02	0.14	44.13
Happiness	EM	18	− 0.001	-0.11	0.11	0.998		0.008	0.09	19.93
Happiness	IPS	18	0.003	-0.17	0.18	0.966		0.06	0.26	66.33
Neutral	Executive	30	0.004	-0.26	0.27	0.978		0.08	0.28	72.48
Neutral	EM	14	0.20	-0.13	0.49	0.237		0.11	0.33	69.92
Neutral	IPS	10	0.004	-0.42	0.43	0.986		0.24	0.49	84.77
Sadness	Executive	70	0.09	0.01	0.17	0.02	*	0.02	0.13	38.50
Sadness	EM	17	0.10	-0.06	0.27	0.218		0.03	0.18	50.73
Sadness	IPS	18	0.17	0.07	0.26	< 0.001	***	0.009	0.10	20.84
Surprise	Executive	43	0.10	0.02	0.17	0.01	*	0.01	0.10	28.01
Surprise	EM	10	0.10	-0.09	0.29	0.305		0.02	0.15	43.77
Surprise	IPS	14	0.11	0.03	0.20	0.01	*	0.001	0.001	0.001
Total	Executive	83	0.22	0.16	0.28	< 0.001	***	0.009	0.096	23.28
Total	EM	21	0.34	0.22	0.45	< 0.001	***	0.02	0.15	32.04
Total	IPS	31	0.29	0.21	0.36	< 0.001	***	0.012	0.11	25.95

Note. * *p* < .05; ** *p* < .01; *** *p* < .001. CI = Confidence Interval. EM = Episodic Memory; IPS = Information Processing Speed. “Total” rows refer to studies that reported global emotion recognition scores (aggregated across emotions).

#### Overall heterogeneity and publication bias

Between-study heterogeneity was assessed using the estimated variance component ($\:\tau\:$^2^), its square root ($\:\tau\:$), and the proportion of variance attributable to heterogeneity (*I*^2^). The number of included effects per combination ranged from 10 to 86. Heterogeneity varied widely across combinations, with *I*^2^ values ranging from 0% to 84.77%, indicating large differences in effect consistency depending on the emotion and cognitive domain considered.

Assessment of overall publication bias, including funnel plots and statistical tests, is presented in Supplementary Material (Section S5).

### Subgroup analyses: emotion–cognition associations by neurocognitive domain

#### Emotion–cognition associations within the executive function domain

##### Subdomain-level effects within executive functioning

We conducted a subgroup multilevel meta-analysis focusing on the Executive Function domain. The subdomain (e.g., abstraction, attention, flexibility, fluency, inhibition, and working memory) was included as a categorical moderator to assess whether the association between emotion and cognitive performance differed across executive components. The model estimated the strength of association using Fisher’s *z*-transformed effect sizes and included a random intercept for study ID to account for within-study clustering. Forest plots can be found in Supplementary Material (Section S9).

Predicted effect sizes for each emotion–executive subdomain pairing were reported in Table 2. Out of the 46 tested combinations between emotions and executive subdomains, 17 yielded statistically significant associations (*p* < .05), with most effects falling within the small-to-moderate range.

In the Abstraction subdomain, only a significant positive effect was observed for Total Score (*r* = .24, CI_95%_ [0.14, 0.33], *p* < .001). Within Fluency, associations emerged for Disgust (*r* = .13, CI_95%_ [0.02, 0.23], *p* = .02), Fear (*r* = .17, CI_95%_ [0.09, 0.26], *p* < .001), Sadness (*r* = .12, CI_95%_ [0.03, 0.20], *p* = .007), Surprise (*r* = .13, CI_95%_ [0.04, 0.23], *p* = .006), and Total Score (*r* = .24, CI_95%_ [0.14, 0.33], *p* < .001). Significant effects were also observed in Inhibition for Total Score (*r* = .24, CI_95%_ [0.10, 0.36], *p* < .001) with a trend-level association found for Disgust (*r* = .12, CI_95%_ [-0.004, 0.24], *p* = .058). The Working Memory subdomain showed significant links with Anger (*r* = .15, CI_95%_ [0.06, 0.23], *p* < .001), Fear (*r* = .22, CI_95%_ [0.10, 0.34], *p* < .001), Disgust (*r* = .17, CI_95%_ [0.06, 0.28], *p* = .001), Sadness (*r* = .17, CI_95%_ [0.03, 0.30], *p* = .01), and Total Score (*r* = .21, CI_95%_ [0.14, 0.29], *p* < .001).

Additionally, an unexpected negative association between Happiness and Flexibility (*r* = − .18, CI_95%_ [-0.30, − 0.06], *p* = .003) was found, suggesting that reduced executive flexibility was linked to better recognition of happy expressions. This pattern was not observed for other emotions, which mostly showed non-significant and directionally opposite trends.

Overall, Fear, Disgust, and Sadness showed the most consistent associations with executive components. In contrast, Neutral and Happy stimuli rarely yielded significant effects.

Among subdomains, Fluency and Working Memory showed the strongest and most reliable links with emotion recognition, while Inhibition and Flexibility tended to show weaker and non-significant associations. Full statistics and confidence intervals are reported in Table 2.

**Table 2:** Overall associations between emotion recognition and cognitive performance Within Executive Subdomains, with heterogeneity indices.

Emotion type	Sub-domain	k	*r*	Lower CI	Upper CI	*p*		$\:\tau\:$ ^2^	$\:\tau\:$	I^2^
Anger	Abstraction	12	0.080	-0.086	0.242	0.342		0.044	0.209	61.83
Anger	Attention	3	0.128	-0.322	0.531	0.584		0.133	0.364	79.08
Anger	Flexibility	8	0.032	-0.094	0.157	0.620		0.004	0.061	11.49
Anger	Fluency	24	0.100	-0.03	0.227	0.131		0.040	0.201	59.36
Anger	Inhibition	10	0.09	-0.023	0.179	0.129		0.000	0.001	0.001
Anger	Working memory	13	0.148	0.064	0.230	< 0.001	***	0.000	0.001	0.001
Disgust	Abstraction	9	0.028	-0.141	0.195	0.749		0.032	0.180	55.44
Disgust	Flexibility	5	0.077	-0.068	0.219	0.296		0.000	0.001	0.001
Disgust	Fluency	20	0.126	0.016	0.232	0.024	*	0.019	0.137	41.13
Disgust	Inhibition	7	0.119	-0.004	0.239	0.058	✝	0.000	0.001	0.001
Disgust	Working memory	9	0.173	0.065	0.277	0.001	**	0.003	0.050	8.44
Fear	Abstraction	12	0.066	-0.066	0.196	0.329		0.021	0.146	44.09
Fear	Attention	3	-0.062	-0.285	0.167	0.598		0.009	0.097	21.1
Fear	Flexibility	8	0.099	-0.014	0.209	0.085	✝	0.000	0.001	0.001
Fear	Fluency	24	0.177	0.087	0.265	< 0.001	***	0.013	0.115	32.43
Fear	Inhibition	10	0.087	-0.085	0.254	0.32		0.036	0.190	55.2
Fear	Working memory	12	0.224	0.102	0.338	< 0.001	***	0.013	0.115	30.99
Happiness	Abstraction	11	-0.007	-0.163	0.149	0.9322		0.033	0.182	57.74
Happiness	Attention	3	0.065	-0.286	0.401	0.724		0.067	0.258	65.53
Happiness	Flexibility	8	-0.182	-0.297	-0.062	0.003	**	0.003	0.050	8.02
Happiness	Fluency	23	-0.044	-0.174	0.086	0.507		0.039	0.197	60.51
Happiness	Inhibition	11	-0.03	-0.155	0.096	0.642		0.011	0.104	24.7
Happiness	Working memory	13	0.098	-0.047	0.239	0.186		0.024	0.155	45.19
Neutral	Abstraction	3	-0.068	-0.819	0.769	0.902		0.588	0.767	95.25
Neutral	Attention	3	-0.151	-0.516	0.261	0.477		0.103	0.320	74.54
Neutral	Flexibility	5	0.012	-0.169	0.193	0.896		0.010	0.100	27.77
Neutral	Fluency	9	-0.113	-0.486	0.295	0.596		0.156	0.395	82.28
Neutral	Inhibition	6	0.003	-0.323	0.328	0.986		0.094	0.307	77.03
Neutral	Working memory	4	-0.056	-0.286	0.179	0.641		0.014	0.118	29.38
Sadness	Abstraction	12	0.033	-0.116	0.180	0.668		0.031	0.177	53.92
Sadness	Attention	3	-0.010	-0.325	0.306	0.950		0.049	0.222	58.53
Sadness	Flexibility	8	0.113	-0.021	0.243	0.098	✝	0.007	0.081	18.61
Sadness	Fluency	23	0.119	0.032	0.203	0.007	**	0.010	0.101	28.87
Sadness	Inhibition	10	0.039	-0.077	0.155	0.508		0.005	0.073	15.32
Sadness	Working memory	13	0.172	0.035	0.303	0.014	*	0.021	0.145	41.75
Surprise	Abstraction	9	0.044	-0.135	0.221	0.632		0.039	0.197	59.71
Surprise	Flexibility	4	0.046	-0.113	0.204	0.569		0.000	0.001	0.001
Surprise	Fluency	17	0.135	0.037	0.231	0.007	**	0.010	0.100	30.14
Surprise	Inhibition	6	0.030	-0.173	0.232	0.771		0.028	0.169	49.28
Surprise	Working memory	6	0.217	0.011	0.406	0.039	*	0.031	0.175	52.91
Total	Abstraction	13	0.241	0.143	0.335	< 0.001	***	0.008	0.087	21.12
Total	Attention	5	0.269	0.128	0.399	< 0.001	***	0.000	0.001	0.001
Total	Flexibility	6	0.143	0.008	0.273	0.037	*	0.000	0.001	0.001
Total	Fluency	32	0.239	0.137	0.335	< 0.001	***	0.022	0.148	41.8
Total	Inhibition	11	0.237	0.101	0.365	< 0.001	***	0.016	0.128	32.65
Total	Working memory	15	0.215	0.137	0.291	< 0.001	***	0.001	0.023	1.85

Note. * *p* < .05; ** *p* < .01; *** *p* < .001; ✝ marginal (*p* < .10). CI = Confidence Interval. For consistency of interpretation, correlation signs for time-based tasks (e.g., Stroop, TMT) in the Flexibility and Inhibition domains were reversed so that positive correlations uniformly reflect better cognitive performance. “Total” rows refer to studies that reported global emotion recognition scores (aggregated across emotions).

##### Heterogeneity within executive subdomains and publication bias

Between-study heterogeneity was assessed using the estimated variance component ($\:\tau\:$^2^), its square root ($\:\tau\:$), and the proportion of variance attributable to heterogeneity (*I*^2^ see Supplementary Material, Section S3). The number of included effects per model ranged from 3 to 32. Heterogeneity varied substantially across combinations, with *I²* values ranging from near 0 to over 90%, suggesting meaningful differences in the consistency of effects across emotional and executive subdomain.

Low *I*^2^ values (close to 0%) observed for some combinations likely reflect either true consistency in effect sizes across studies or limited variability due to the small number of included comparisons, and/or the potential inclusion of multiple effects from the same articles, which was accounted for in the multilevel model. These values should thus be interpreted with caution.

Assessment of publication bias, including funnel plots and statistical tests, is presented in Supplementary Material (Section S6).

#### Emotion–cognition associations within the episodic memory domain

##### Subdomain-level effects within the episodic memory domain

We conducted a subgroup multilevel meta-analysis focusing on the Episodic Memory Domain. The subdomain (Verbal versus Visual) was included as a categorical moderator to assess whether the association between emotion and cognitive performance varied across measures. The model estimated the strength of association using Fisher’s *z*-transformed effect sizes and included a random intercept for study ID to account for within-study clustering. Forest plots can be found in Supplementary Material (Section S9).

Predicted effect sizes for each emotion–task pairing (verbal vs. visual) are reported in Table 3. Among the 16 emotion–subdomain combinations tested, 5 showed statistically significant associations (*p* < .05), and one additional effect reached trend level (*p* < .10), with most estimates falling within the small-to-moderate range.

For Verbal Episodic Memory, significant positive associations were observed for Disgust (*r* = .17, CI_95%_ [0.08, 0.29], *p* = .005), and Total (*r* = .31, CI_95%_ [0.21, 0.40], *p* < .001). A trend-level associations was also observed for Fear (*r* = .19, CI_95%_ [-0.01, 0.39], *p* = .06).

For Visual Episodic Memory, significant positive associations emerged for Disgust (*r* = .15, CI_95%_ [0.01, 0.28], *p* = .03), Fear (*r* = .31, CI_95%_ [0.05, 0.53], *p* = .02) and Total (*r* = .43, CI_95%_ [0.16, 0.63], *p* = .002).

Overall, Visual Episodic Memory yielded more frequent and robust associations than Verbal Episodic Memory, particularly for negative emotions such as Disgust and Fear. Associations with Anger, Happiness, Sadness, Surprise, and Neutral stimuli remained weak and non-significant across both tasks. Full statistics and confidence intervals are reported in Table 3.

**Table 3:** Overall associations between emotion recognition and cognitive performance Within Episodic Memory tasks, with heterogeneity indices.

Emotion type	Sub-domain	k	*r*	Lower CI	Upper CI	*p*		$\:\tau\:$ ^2^	$\:\tau\:$	I^2^
Anger	Verbal	11	0.122	-0.089	0.3229	0.256		0.05906	0.2430	59.23
Anger	Visual	10	0.198	-0.0929	0.4588	0.180		0.11296	0.3360	72.93
Disgust	Verbal	8	0.173	0.0513	0.2907	0.005	**	0.0001	0.0001	0.0001
Disgust	Visual	7	0.148	0.0158	0.2764	0.028	*	0.0001	0.0001	0.0001
Fear	Verbal	11	0.199	-0.0125	0.3943	0.064	✝	0.0610	0.2471	60.05
Fear	Visual	10	0.306	0.0469	0.5275	0.021	*	0.09135	0.3022	68.54
Happiness	Verbal	9	− 0.008	-0.1807	0.1637	0.921		0.02712	0.1646	46.54
Happiness	Visual	9	0.029	-0.0848	0.1423	0.616		0.0001	0.0001	0.0001
Neutral	Verbal	7	0.090	-0.2059	0.3717	0.553		0.07066	0.2658	60.61
Neutral	Visual	7	0.295	-0.0841	0.6003	0.124		0.13421	0.3663	74.51
Sadness	Verbal	9	0.122	-0.0304	0.2696	0.116		0.01683	0.1297	35.07
Sadness	Visual	8	0.097	-0.1249	0.3101	0.391		0.04575	0.2138	59.16
Surprise	Verbal	5	0.119	-0.162	0.3831	0.406		0.04563	0.2136	61.9
Surprise	Visual	5	0.102	-0.0397	0.2399	0.150		0.0001	0.0001	0.0001
Total	Verbal	12	0.306	0.209	0.397	< 0.001	***	0.0001	0.0001	0.0001
Total	Visual	8	0.426	0.1663	0.6317	0.0019	**	0.08308	0.2882	63.82

Note. * *p* < .05; ** *p* < .01; *** *p* < .001; ✝ marginal (*p* < .10). CI = Confidence Interval. “Total” rows refer to studies that reported global emotion recognition scores (aggregated across emotions).

##### Heterogeneity within episodic memory subdomains and publication bias

Between-study heterogeneity was assessed using the estimated variance component ($\:\tau\:$^2^), its square root ($\:\tau\:$), and the proportion of variance attributable to heterogeneity (*I*^2^, see Supplementary Material, Section S3). The number of included effects per model ranged from 5 to 11. Heterogeneity varied substantially across combinations, with *I²* values ranging from 0% to 74.51%, indicating low to moderate heterogeneity across emotion and episodic memory subdomains.

Assessment of publication bias, including funnel plots and statistical tests, is presented in Supplementary Material (Section S7).

#### Emotion–cognition associations within the processing speed domain

##### Subdomain-level effects within IPS tasks

We conducted a subgroup multilevel meta-analysis focusing on the IPS domain. The type of task (SDMT versus PASAT) was included as a categorical moderator to assess whether the association between emotion and cognitive performance varied across measures. The model estimated the strength of association using Fisher’s *z*-transformed effect sizes and included a random intercept for study ID to account for within-study clustering. Forest plots can be found in Supplementary Material (Section S9).

Predicted effect sizes for each emotion–task pairing (SDMT versus PASAT) are reported in Table 4. Among the 16 emotion–subdomain combinations tested, 8 showed statistically significant associations (*p* < .05), and one additional effect reached trend level (*p* < .10), with most estimates falling within the small-to-moderate range.

For SDMT, significant positive associations were observed for Anger (*r* = .18, CI_95%_ [0.06, 0.30], *p* = .004), Disgust (*r* = .15, CI_95%_ [0.05, 0.25], *p* = .003), Fear (*r* = .20, CI_95%_ [0.07, 0.32], *p* = .002), Sadness (*r* = .16, CI_95%_ [0.04, 0.27], *p* = .009), Surprise (*r* = .13, CI_95%_ [0.03, 0.22], *p* = .009), and Total score (*r* = .29, CI_95%_ [0.23, 0.3635], *p* < .001). No significant associations were observed for Neutral or Happiness stimuli on SDMT performance.

For PASAT, significant positive effects emerged for Neutral (*r* = .24, CI_95%_ [0.04, 0.41], *p* = .004), Sadness (*r* = .21, CI_95%_ [0.07, 0.35], *p* = .005), and Total score (*r* = .26, CI_95%_ [0.14, 0.37], *p* < .001). A trend-level association was also observed for Fear (*r* = .20, CI_95%_ [-0.01, 0.39], *p* = .06).

Overall, SDMT yielded a slightly higher number of statistically significant associations than PASAT. Notably, only SDMT showed reliable effects for Anger and Disgust, while both tasks revealed significant associations for Fear, Sadness and Surprise. However, the strength of the associations (*r*) was generally comparable across tasks. Full statistics and confidence intervals are reported in Table 4.

**Table 4:** Overall associations between emotion recognition and cognitive performance Within IPS tasks, with heterogeneity indices.

Emotion type	Sub-domain	k	*r*	Lower CI	Upper CI	*p*		$\:\tau\:$ ^2^	$\:\tau\:$	*I* ^2^
Anger	PASAT	7	0.1318	-0.0848	0.3365	0.2324		0.03489	0.18678	40.25
Anger	SDMT	13	0.1815	0.0557	0.3016	0.0049	**	0.02285	0.15117	38.63
Disgust	PASAT	5	0.1106	-0.0766	0.2902	0.2464		0.0001	0.0001	0.0001
Disgust	SDMT	11	0.1541	0.0509	0.254	0.0035	**	0.0044	0.06633	10.67
Fear	PASAT	7	0.196	-0.0123	0.3879	0.0649	✝	0.03076	0.1754	37.27
Fear	SDMT	13	0.1971	0.0705	0.3173	0.0024	***	0.02372	0.15402	39.49
Happiness	PASAT	6	-0.082	-0.2487	0.0893	0.3483		0.00798	0.08934	16.04
Happiness	SDMT	12	0.0172	-0.1693	0.2025	0.8574		0.07396	0.27196	71.14
Neutral	PASAT	4	0.2358	0.0453	0.4098	0.0157	*	0.0001	0.0001	0.0001
Neutral	SDMT	6	0.0306	-0.4459	0.4935	0.9065		0.30059	0.54826	88.64
Sadness	PASAT	6	0.2152	0.0659	0.355	0.005	**	0.0001	0.0001	0.0001
Sadness	SDMT	12	0.16	0.0403	0.2751	0.009	**	0.01675	0.12943	34.09
Surprise	PASAT	5	0.0558	-0.1239	0.232	0.5439		0.0001	0.0001	0.0001
Surprise	SDMT	9	0.1285	0.0312	0.2234	0.0097		0.0001	0.0001	0.0001
Total	PASAT	10	0.2609	0.1399	0.3741	< 0.001	***	0.0116	0.1077	22.9
Total	SDMT	21	0.2889	0.2283	0.3473	< 0.001	***	0.0001	0.0001	0.0001

Note. * *p* < .05; ** *p* < .01; *** *p* < .001; ✝ marginal (*p* < .10). CI = Confidence Interval.

##### Heterogeneity within IPS tasks and publication bias

Between-study heterogeneity was assessed using the estimated variance component ($\:\tau\:$^2^), its square root ($\:\tau\:$), and the proportion of variance attributable to heterogeneity (*I*², see Supplementary Material, Section S3). The number of included effects per model ranged from 4 to 22. Heterogeneity varied substantially across combinations, with *I²* values ranging from 0% to 88.64%, indicating low to substantial heterogeneity across emotion and IPS subdomains.

Assessment of publication bias, including funnel plots and statistical tests, is presented in Supplementary Material (Section S8).

### Clinical and demographic moderators: meta-regressions

Meta-regressions examining moderators across combinations of emotional recognition and cognitive domains revealed several significant effects. Both clinical and demographic variables (including disability level, disease duration, and mood symptoms), differentially influenced the relationship between emotional recognition and cognitive domains, with patterns varying depending on the emotion involved.

Age emerged as a negative moderator for the correlation between neutral emotion recognition and episodic memory (*r*_change_ = − 0.101), indicating a decrease in the strength of this association with increasing age.

Depression levels also moderated the relationships involving happiness: higher depression was linked to weaker associations between recognition of happiness and both executive (*r*_change_ = − 0.190) and episodic memory domains (*r*_change_ = − 0.215).

Anxiety appeared to enhance the strength of the correlation between total emotion recognition and executive function (*r*_change_ = 0.128), whereas fatigue was associated with a weaker link between disgust recognition and executive performance (*r*_change_ = − 0.137).

Higher disability levels, as indexed by EDSS scores, were associated with stronger correlations between emotion recognition and cognitive performance in multiple contexts. Specifically, this was observed for neutral expressions in relation to both information processing speed (*r*_change_ = 0.750) and executive functions (*r*_change_ = 0.467), as well as for sadness (*r*_change_ = 0.117), surprise (*r*_change_ = 0.09), disgust (*r*_change_ = 0.09), and happiness (*r*_change_ = 0.11) in association with executive functions. A significant effect was also found for surprise and episodic memory (*r*_change_ = 0.512), suggesting that disability may enhance the relationship between certain emotional and cognitive domains.

Conversely, longer disease duration was generally associated with weaker correlations, notably between episodic memory and recognition of neutral (*r*_change_ = − 0.165), anger (*r*_change_ = − 0.085), fear (*r*_change_ = − 0.08), and total score (*r*_change_ = − 0.040). Interestingly, this trend reversed for the combination of surprise and episodic memory, where a longer disease duration predicted a stronger correlation (*r*_change_ = 0.448).

Lastly, phenotype influenced outcomes: studies focusing exclusively on RRMS patients showed a weaker association between happiness recognition and executive functions compared to those with mixed samples (*r*_change_ = − 0.184). Full statistics and confidence intervals are reported in Supplementary Table S8.

## Discussion

### Main findings

The present meta-analysis aimed to investigate the association between emotion recognition and cognitive functioning in pwMS. Specifically, we examined whether emotion recognition performance was correlated with neurocognition, specifically executive functions, episodic memory, and IPS. Additionally, we explored potential moderators that could account for heterogeneity across studies, including clinical and demographic factors.

Our results indicated statistically significant associations, although the strength and consistency of this relationship varied across cognitive domains. All emotions and subdomains combined, effect sizes were small to moderate across domains, with slightly higher pooled associations for episodic memory (*r* = .17) and IPS (*r* = .16), and a smaller effect for executive functioning (*r* = .11). These modest correlations are consistent with the presence of heterogeneous emotion–cognition relationships depending on the specific subdomain, task or emotion involved.

When examined by cognitive domain, significant positive associations were found across all three domains. The strongest and most consistent associations were found with processing speed and executive functioning. While emotion recognition was significantly associated with episodic memory, this relationship appeared to be more emotion-specific compared to processing speed or executive functioning, with stronger effects for emotions like fear and disgust. These domain-level patterns should nevertheless be interpreted with caution given the heterogeneity observed across studies, suggesting that the reported associations reflect overall trends rather than fully consistent effects.

Overall, recognition of negative emotions (particularly fear, sadness, and anger) was more strongly associated with cognitive performance than the recognition of positive emotions such as happiness or neutral expressions.

Meta-regression analyses further revealed that several clinical and demographic variables significantly moderated the strength of the observed associations. In particular, EDSS score, disease duration, depression, and fatigue were identified as relevant moderators in specific domain-by-emotion combinations.

### Emotion–cognition associations across cognitive domains and tasks

#### Emotion recognition and executive functions

Analyses conducted at the subdomain level revealed important differences in the strength of emotion–cognition associations, reinforcing the idea that not all cognitive operations are equally related to emotion recognition.

Within the executive domain, verbal fluency and working memory consistently showed stronger and more reliable associations with emotion recognition, whereas inhibition and flexibility produced weaker or non-significant effects. This heterogeneity may reflect the varying degrees to which these executive components support facial emotion recognition.

Verbal fluency and working memory both rely on broad frontal-subcortical networks and engage multiple executive functions in parallel, including controlled retrieval, attentional shifting, and information updating^44^. In MS, these two functions are among the most frequently impaired and are often used as sentinel markers of global cognitive dysfunction^45^. Verbal fluency deficits, in particular, emerge early in the disease course and reflect a combination of lexical access, processing speed, and cognitive flexibility^46^. Working memory impairments, on the other hand, are typically linked to fronto-parietal dysfunction and are believed to disrupt the maintenance and manipulation of socially relevant information^47^.

Neuroimaging evidence further supports the involvement of prefrontal regions in emotion recognition performance. In pwMS, reduced activation of the left ventrolateral prefrontal cortex (vlPFC) and anterior insula has been observed during tasks requiring the recognition of unpleasant emotions such as sadness, fear, and anger^48^. Similarly, studies in patients with traumatic brain injury have shown that damage to the ventromedial prefrontal cortex leads to impairments in the recognition of negative emotions^49^.

Overall, these findings suggest that executive subcomponents most reliant on broader frontal-subcortical integrity (such as verbal fluency and working memory) are implicated in facial emotion recognition, while more circumscribed functions like inhibition or flexibility may be insufficient, on their own, to support performance in this domain. It is also worth noting that the weaker associations observed for flexibility may also reflect the nature of the tasks commonly used to assess this function. Standard measures such as the TMT capture shifting with a strong motor component, which may not fully reflect more complex or spontaneous forms of cognitive flexibility involved in social cognition.

#### Emotion recognition, IPS, and memory: the role of visual processing

Within the processing speed domain, both the SDMT and the PASAT showed positive associations with emotion recognition, but their patterns differed in selectivity rather than magnitude. The SDMT yielded significant effects across a broader range of emotions (including anger, disgust, fear, and sadness), suggesting stronger links with emotional stimuli, particularly those with negative valence. In contrast, the PASAT showed significant associations with fewer emotions (mainly fear and sadness). Interestingly, neutral expressions were significantly linked with PASAT but not SDMT. This specific association between PASAT and neutral expressions may reflect the greater ambiguity of neutral stimuli, which could require more elaborative and sustained processing, mechanisms more closely aligned with working memory demands.

One possible explanation for the stronger and more consistent associations observed with the SDMT lies in its visual and symbol-based nature. This task primarily relies on visuo-perceptual processing and rapid cross-modal matching, which are also fundamental to decoding facial emotional expressions. By engaging perceptual and attentional systems involved in the rapid identification and integration of visual cues, the SDMT may tap into neural networks that overlap more directly with those required for facial emotion recognition. In contrast, the PASAT is an abstract, auditory-based task, that primarily taxes sustained attention and working memory, with limited reliance on visual-perceptual mechanisms that are critical for facial emotion recognition^50^.

A comparable pattern of task-specificity was observed within the episodic memory domain: interestingly, visual tasks yielded more frequent and stronger associations with emotion recognition than verbal memory tasks. This discrepancy may also be due to the perceptual overlap between facial emotion recognition and the type of encoding involved in visual memory paradigms, which likely recruit overlapping neural substrates such as occipito-temporal and limbic regions^51–53^. In contrast, verbal memory may rely on more distinct, left-lateralized language-based systems, less directly engaged in processing emotional facial cues^54^.

Multiple neuroimaging studies in MS support the notion of a disrupted visuo-limbic network underlying facial emotion recognition deficits. Studies have shown that impairments in facial emotion recognition are not solely related to general cognitive decline but are instead linked to structural and functional alterations in a network that includes both visual and limbic regions. For instance, Mike et al. (2013)^55^ demonstrated that deficits in recognizing emotional expressions were associated with cortical thickness in the fusiform gyrus, a region crucial for face perception, and to disconnection of occipito-temporal and fronto-limbic white matter tracts (see also^21,56^. Similarly, Golde et al. (2020)^26^ reported that poorer emotion recognition was associated with lower resting-state functional connectivity of the right fusiform gyrus with the right lateral occipital cortex. Pfaff et al. (2019)^57^ also reported trend-level activation in the amygdala and fusiform gyrus during negative emotion processing in MS. Such data argue that effective emotion recognition in MS seems to depend on the integrity and functional interactions of a visuo‑limbic network, rather than on limbic structures alone.

Notably, this pattern does not reflect a reorganization unique to MS, but rather a disruption of the same visuo-limbic network known to support facial emotion recognition in healthy individuals. Functional neuroimaging in non-clinical populations has shown that successful decoding of emotional expressions engages both visual-perceptual areas (e.g., fusiform gyrus, superior temporal sulcus) and emotion-sensitive regions such as the amygdala^51,52^.

### Emotion valence seems to be a modulating factor

#### Methodological factors

The stronger and more consistent associations observed for negatively valenced emotions should also be interpreted in light of potential measurement-related factors. Indeed, recognition of positive emotions, particularly happiness, is typically associated with high accuracy levels in both clinical and non-clinical populations^58–61^, which may lead to reduced variability in performance and limit the ability to detect correlations with cognitive measures. In MS specifically, recent work has also raised the possibility that the apparent preservation of positive emotion recognition may be partly related to methodological characteristics of the tasks used, rather than reflecting a fully preserved process per se^62^.

In contrast, negative emotional expressions are generally more difficult to recognize and tend to show greater inter-individual variability, especially in pwMS, where deficits have been more consistently reported for these emotions^9,19^. This increased variability may contribute to the emergence of detectable associations with cognitive performance in the present meta-analysis.

In this sense, the current findings are broadly consistent with previous meta-analyses reporting more pronounced impairments in the recognition of negative emotions in MS^9,18,19^.The present study extends this literature by suggesting that these dimensions may also be those in which the relationship between emotion recognition and cognitive functioning is most readily observable. However, it is important to note that weaker associations for positive emotions should not be interpreted as evidence of absence of a relationship, but may instead reflect methodological constraints, such as ceiling effects in task performance.

#### Perceptual and motivational factors

Interestingly, the emotion of happiness displayed an atypical pattern. While generally yielding weak or non-significant associations with cognition, happiness was negatively associated with executive flexibility. Because scores on time-based tasks were reversed for consistency across all cognitive measures, this counterintuitive finding suggests that individuals who exhibited reduced cognitive flexibility (i.e., longer response times) performed better on tasks involving recognition of happy expressions. This effect was not observed for any other emotion.

One possible explanation for the paradoxical finding observed for happiness is that this emotion, due to its high prototypicality and distinctive perceptual features^58^, may be processed via more automatic and perceptual mechanisms than other emotions. Behavioral studies have consistently shown that happy faces are recognized faster and more accurately than other emotional expressions in explicit recognition tasks^58,63^, likely because of their salience, symmetry, and low ambiguity. Such evidence suggests that the recognition of positive emotions can engage fast, bottom-up perceptual pathways that are relatively independent of top-down executive control. In this context, individuals with reduced cognitive flexibility, typically characterized by limited shifting between cognitive operations, might actually benefit from relying on these more direct, stimulus-driven processes, which are sufficient for identifying clearly decodable emotions like happiness. In contrast, more ambiguous or context-dependent negative emotions such as fear or anger are often more cognitively demanding to interpret, requiring higher-order operations, including the ability to manage and manipulate relevant information to resolve ambiguity. This may explain why the paradoxical association was observed only for happiness, and not for other emotional categories.

An alternative explanation may lie in motivational models of emotional processing, such as Socioemotional Selectivity Theory (SST^64^. According to SST, individuals who perceive their future as constrained tend to shift their cognitive priorities toward emotionally meaningful and rewarding stimuli, typically those with positive valence^65,66^. This motivational shift, originally described in the context of aging, has also been observed in other populations facing uncertainty or perceived loss of resources^67–71^. In the context of MS, where disease progression is unpredictable and cognitive decline may be anticipated, individuals may adopt similar prioritization strategies^72^. Patients with reduced executive flexibility may be particularly susceptible to such a shift, relying more heavily on low-effort, emotionally rewarding content. In this framework, enhanced recognition of happy expressions among pwMS with lower flexibility would not reflect preserved cognitive capacity, but rather a selective engagement with positive cues driven by motivational factors. This interpretation aligns with the idea that positivity biases can emerge adaptively in response to perceived constraints, and may influence emotion processing across patients depending on their illness perception and subjective time horizon.

While both interpretations offer plausible accounts for the specific association between happiness and reduced flexibility in pwMS, this result, although intriguing, remains isolated and requires further validation. Executive flexibility was the only domain to show a significant association with happiness; all other executive domains (including working memory) yielded non-significant correlations despite similarly negative trends (all *r* values ranging from − 0.18 to − 0.006, with the exception of abstraction and working memory, which were slightly positive). The lack of consistent findings, particularly with working memory, challenges the idea of a generalizable or robust link between happiness recognition and executive functioning.

#### The specific case of surprise

The pattern observed for surprise deserves specific consideration. In the present meta-analysis, surprise showed some positive associations with cognitive performance, particularly within executive functioning and processing speed, but these effects were less consistent and generally weaker than those observed for negatively valenced emotions.

One possible explanation lies in the inherent ambiguity of surprise as an emotional category. Unlike other basic emotions, surprise does not have a fixed valence and may be interpreted as positive, negative, or neutral depending on contextual cues^73^. This ambiguity may reduce the consistency of its operationalization across studies and tasks, leading to increased heterogeneity and less stable associations with cognitive measures.

In addition, the recognition of surprise may rely more heavily on contextual inference and integration processes than on the direct decoding of distinctive perceptual features. In many experimental paradigms, surprise is presented in isolation, without contextual information, which may introduce variability in how participants interpret and categorize these expressions. This variability may, in turn, attenuate the strength and consistency of observed correlations with cognitive performance.

From a measurement perspective, the variability associated with surprise contrasts with the relative stability observed for both highly prototypical positive emotions (e.g., happiness) and more consistently impaired negative emotions (e.g., fear, disgust). As a result, surprise may occupy an intermediate position, where some associations with cognition can be detected, but with reduced robustness across studies and cognitive domains.

Overall, the present findings suggest that the relationship between surprise recognition and cognitive functioning in MS is likely to be more context-dependent and sensitive to task characteristics than for other emotional categories. Future studies would benefit from going beyond accuracy scores alone and examining how participants subjectively interpret the valence of surprise expressions (e.g., positive, negative, or neutral). Such an approach may help clarify whether individual differences in valence attribution contribute to the variability of surprise-cognition associations observed across studies.

Moreover, facial emotion recognition paradigms often require participants to select a label within a forced-choice format. In this context, when participants experience difficulty recognizing fear, they frequently default to selecting surprise, thereby masking impairments in fear recognition. Thus, depending on the paradigm employed, the associations with cognitive functioning may vary. The inclusion of a systematic error analysis, including intra-individual error-profile approaches, could provide valuable insights into whether emotion recognition impairments in MS are emotion-specific or reflect broader patterns of confusion across emotional categories.

### Links with clinical and demographic characteristics

Meta-regression analyses revealed that certain clinical and demographic variables moderated the strength of emotion–cognition associations in pwMS. Specifically, studies involving patients with higher disability scores (EDSS) tended to show stronger associations between emotion recognition and cognitive performance, suggesting that emotional decoding difficulties may become more tightly coupled to cognitive decline in more clinically severe profiles.

In contrast, disease duration showed a limited and emotion-specific influence, with a significant negative effect found only for neutral expressions in relation to episodic memory.

The impact of mood-related symptoms was also circumscribed. Although depression is typically associated with generalized cognitive slowing and emotional blunting, its moderating effect was limited to a reduced association between cognitive performance and the recognition of happiness. This finding may reflect the fact that positive emotional expressions are harder to process in individuals experiencing depressive symptoms. Anxiety showed a similarly restricted pattern, with a small but significant effect limited to the total emotion recognition score in relation to executive functioning. This isolated effect might suggest that anxiety, by increasing arousal or vigilance, could enhance the recruitment of executive resources during emotion recognition tasks.

The limited effects observed for mood-related variables may partly reflect methodological factors. First, many of the included studies applied exclusion criteria for moderate to severe depression and anxiety, thereby reducing variability and potentially underestimating their true impact. Second, heterogeneity in assessment tools may have further obscured existing associations. For instance, studies used both the full Beck Depression Inventory (BDI) and the abbreviated BDI-FS. As previously discussed^74^, the full BDI may yield inflated scores in pwMS due to overlapping symptoms with the disease itself (such as fatigue or sleep difficulties) which are not filtered out in the total score. This symptom overlap may dilute the specificity of the depression-emotion recognition link and contribute to inconsistent findings across studies.

These findings may be clinically relevant given that both anxiety and depression are frequently underdiagnosed and undertreated in pwMS, despite their known impact on quality of life, emotional processing and cognitive functioning. Although our results revealed only selective effects, they suggest that mood symptoms can shape how cognitive and emotional processes interact in MS.

No consistent moderating effects were found for age, fatigue, or alexithymia. Although a few isolated associations reached significance, these variables were only available in a subset of studies, limiting interpretability. Future research with more consistent measurement of these variables is needed to clarify their role.

### Limitations and future directions

Although this meta-analysis provides a comprehensive synthesis of the relationship between emotion recognition and cognitive functioning in MS, several limitations should be acknowledged. First, an important aspect to consider when interpreting these findings is the substantial heterogeneity observed across studies. As reported in the Results section, the consistency of effect sizes varied widely depending on the emotion and cognitive domain considered, with I^2^ values ranging from low to high levels across combinations. This heterogeneity likely reflects both clinical and methodological variability across studies. From a clinical perspective, differences in disease characteristics (e.g., disease duration, disability level, symptom severity…) may contribute to variability in the strength of emotion–cognition associations. From a methodological standpoint, the use of diverse neuropsychological measures and emotion recognition tasks may also introduce additional variability. Although we attempted to account for part of this heterogeneity through multilevel modeling and meta-regression analyses, residual variability remained. Therefore, the present findings should be interpreted as average tendencies across heterogeneous samples and data rather than as uniform effects, highlighting the need for more standardized approaches in future research.

Second, while these findings support a link between emotion-recognition and cognitive functioning in MS, they remain correlational in nature and cannot establish causality or underlying mechanisms. As such, our results do not confirm whether these deficits reflect a shared substrate or distinct but co-occurring processes. To better understand whether emotion recognition deficits in MS reflect a consequence of general cognitive decline or rely on partially distinct neural mechanisms, future research should combine behavioral, longitudinal, and neuroimaging approaches. Functional neuroimaging studies could offer critical insights into this issue. For instance, tasks probing emotion recognition and executive functioning could be administered during fMRI to assess the degree of overlap or dissociation in activated networks.

Previous studies in MS have already shown that MS patients with impaired emotion recognition exhibit reduced activation in the left ventrolateral prefrontal cortex and anterior insula, particularly during the processing of negative facial expressions^48^. Similarly, reduced activation in the fusiform gyrus and right insula has been observed in MS patients during emotional face processing, even in the absence of behavioral impairments^26^. Another study combining resting-state and diffusion imaging has also reported that reduced connectivity between key socio-cognitive hubs, such as the anterior cingulate cortex and prefrontal regions, was associated with impaired theory of mind in MS^75^.

Comparing activation patterns across cognitive and social-cognitive tasks within the same individuals could help identify whether deficits emerge from a common disruption in fronto-subcortical circuits or from more specific impairments in emotion-related processing pathways. Moreover, studies using multimodal imaging (e.g., combining functional and structural MRI, or diffusion tensor imaging) could clarify whether the same white matter tracts or gray matter regions are implicated in both domains. Mediation models could also test whether processing speed or working memory mediates the impact of MS pathology on emotion recognition performance.

Moreover, longitudinal data could clarify temporal precedence. If cognitive decline is shown to precede and predict subsequent declines in emotion recognition (but not the reverse), this supports a possible causal direction from cognition to social-cognition. Longitudinal data would also allow to compare the strength and direction of reciprocal influences over time. For example, one could test whether baseline deficits in executive functioning, episodic memory, or IPS predict emotion recognition performance at follow-up more strongly than the reverse. Such patterns would offer indirect evidence about likely causal pathways and rule out pure co-occurrence. Longitudinal designs also help identify divergent trajectories across patients: if social cognition declines in some patients independently of general cognition, this argues for dissociable mechanisms. Conversely, if declines consistently co-occur in a temporally ordered fashion (e.g., first IPS, then emotion recognition), this supports the hypothesis of functional interdependence.

However, even longitudinal designs must be interpreted cautiously. Shared variance may still arise from a third variable (e.g., global disease progression, structural damage), and only the integration of multimodal imaging or intervention studies can directly probe mechanistic relationships.

Ultimately, this integrative approach would provide a more precise understanding of the cognitive architecture underlying social cognition in MS, and help determine whether joint remediation strategies are appropriate, or whether distinct interventions should be developed for cognitive and socio-cognitive deficits.

## Conclusion

This meta-analysis contributes to a better understanding of how cognitive and emotional processes may be related in MS. By relying on a multilevel random-effects framework, we addressed the statistical dependencies inherent to meta-analytic data. The distinction between domains and subdomains helped to clarify how specific cognitive functions relate to emotion recognition. Importantly, examining each basic emotion separately provided a clearer picture of their respective cognitive correlates, highlighting patterns that would likely have been masked in global emotion scores.

Emotion recognition and cognitive functioning are moderately related in MS, particularly for negative emotions and cognitive subdomains involving fronto-subcortical circuits. Stronger associations were observed for working memory, verbal fluency, visual memory and IPS. In addition, several clinical and demographic factors modulated the strength of these associations. Among them, disability level (EDSS) emerged as the most consistent moderator, with higher disability levels associated with stronger links between emotion and cognition, especially in the case of negative emotions. These findings suggest partially shared mechanisms between social and nonsocial cognition in MS, influenced by clinical and demographic factors.

Assessing emotional abilities in MS may provide complementary insights into patients’ everyday difficulties, beyond the scope of traditional cognitive assessments. While it is well acknowledged that pwMS often experience difficulties in employment, relationships, and overall quality of life^76^, the specific contribution of cognitive versus emotional impairments to these outcomes remains unclear. We lack precise data on how deficits in emotion recognition or regulation might independently, or jointly, affect social participation or interpersonal stability. Understanding the respective roles of cognitive and emotional impairments is essential for developing interventions that truly address the lived challenges of MS.

## Supplementary Information

Below is the link to the electronic supplementary material.

Supplementary Material 1

## Data Availability

All data generated or analyzed in this study are included in the published article and its supplementary information files. All extracted data (including the summary Excel file) and supplementary result tables are openly available on the Open Science Framework at 10.17605/OSF.IO/3VNA4.

## References

[1] Compston, A. & Coles, A. Multiple sclerosis. *Lancet* **359** (9313), 1221–1231. 10.1016/S0140-6736(02)08220-X (2002). 11955556 10.1016/S0140-6736(02)08220-X

[2] Geurts, J. J. & Barkhof, F. Grey matter pathology in multiple sclerosis. *Lancet Neurol.* **7** (9), 841–851. 10.1016/S1474-4422(08)70191-1 (2008). 18703006 10.1016/S1474-4422(08)70191-1

[3] Noseworthy, J. H., Lucchinetti, C., Rodriguez, M. & Weinshenker, B. G. Multiple sclerosis. *N. Engl. J. Med.* **343** (13), 938–952. 10.1056/NEJM200009283431307 (2000). 11006371 10.1056/NEJM200009283431307

[4] Chiaravalloti, N. D. & DeLuca, J. Cognitive impairment in multiple sclerosis. *Lancet Neurol.* **7** (12), 1139–1151. 10.1016/S1474-4422(08)70259-X (2008). 19007738 10.1016/S1474-4422(08)70259-X

[5] Rao, S. M. et al. Cognitive dysfunction in multiple sclerosis.: II. Impact on employment and social functioning. *Neurology* **41** (5), 692–696. 10.1212/WNL.41.5.692 (1991). 1823781 10.1212/wnl.41.5.692

[6] Prakash, R., Snook, E., Lewis, J., Motl, R. & Kramer, A. Cognitive impairments in relapsing-remitting multiple sclerosis: a meta-analysis. *Mult. Scler. J.* **14** (9), 1250–1261. 10.1177/1352458508095004 (2008). 10.1177/1352458508095004 PMC2847445 18701571

[7] Van Schependom, J. et al. The Symbol Digit Modalities Test as sentinel test for cognitive impairment in multiple sclerosis. *Eur. J. Neurol.* **21** (9), 1219–e72. 10.1111/ene.12463 (2014). 24850580 10.1111/ene.12463

[8] Planche, V., Gibelin, M., Cregut, D., Pereira, B. & Clavelou, P. Cognitive impairment in a population-based study of patients with multiple sclerosis: differences between late relapsing–remitting, secondary progressive and primary progressive multiple sclerosis. *Eur. J. Neurol.* **23** (2), 282–289. 10.1111/ene.12715 (2016). 25903918 10.1111/ene.12715

[9] Cotter, J. et al. Social cognition in multiple sclerosis: a systematic review and meta-analysis. *Neurology* **87** (16), 1727–1736. 10.1212/WNL.0000000000003236 (2016). 27655736 10.1212/WNL.0000000000003236 PMC5085073

[10] Henry, A., Tourbah, A., Chaunu, M. P., Bakchine, S. & Montreuil, M. Social cognition abilities in patients with different multiple sclerosis subtypes. *J. Int. Neuropsychol. Soc.* **23** (8), 653–664. 10.1017/S1355617717000510 (2017). 28656885 10.1017/S1355617717000510

[11] Cecchetto, C. et al. Facial and bodily emotion recognition in multiple sclerosis: the role of alexithymia and other characteristics of the disease. *J. Int. Neuropsychol. Soc.* **20** (10), 1004–1014. 10.1017/S1355617714000939 (2014). 25373767 10.1017/S1355617714000939

[12] Henry, J. D. et al. Evidence for deficits in facial affect recognition and theory of mind in multiple sclerosis. *J. Int. Neuropsychol. Soc.* **15** (2), 277–285. 10.1017/S1355617709090195 (2009). 19203428 10.1017/S1355617709090195

[13] Lenne, B. et al. Impaired recognition of facial emotional expressions in multiple sclerosis. *Neuropsychol. Trends* **2014**, 67–83. (2014).

[14] Jehna, M. et al. An exploratory study on emotion recognition in patients with a clinically isolated syndrome and multiple sclerosis. *Clin. Neurol. Neurosurg.* **112** (6), 482–484. 10.1016/j.clineuro.2010.03.020 (2010). 20399006 10.1016/j.clineuro.2010.03.020

[15] Frith, C. D. Social cognition. *Philos. Trans. R. Soc. B Biol. Sci.* **363** (1499), 2033–2039. 10.1098/rstb.2008.0005 (2008). 10.1098/rstb.2008.0005 PMC2375957 18292063

[16] Henry, J. D., von Hippel, W., Molenberghs, P., Lee, T. & Sachdev, P. S. Clinical assessment of social cognitive function in neurological disorders. *Nat. Rev. Neurol.* **12** (1), 28–39. 10.1038/nrneurol.2015.229 (2016). 26670297 10.1038/nrneurol.2015.229

[17] Bora, E., Özakbaş, S., Velakoulis, D. & Walterfang, M. Social cognition in multiple sclerosis: a meta-analysis. *Neuropsychol. Rev.* **26** (2), 160–172. 10.1007/s11065-016-9320-6 (2016). 27324894 10.1007/s11065-016-9320-6

[18] Roheger, M., Grothe, L., Hasselberg, L., Grothe, M. & Meinzer, M. A systematic review and meta-analysis of socio-cognitive impairments in multiple sclerosis. *Sci. Rep.* **14** (1), 7096. 10.1038/s41598-024-53750-5 (2024). 38528009 10.1038/s41598-024-53750-5 PMC10963773

[19] Lin, X. et al. Social cognition in multiple sclerosis and its subtypes: a meta-analysis. *Mult. Scler. Relat. Disord*. **52**, 102973. 10.1016/j.msard.2021.102973 (2021). 33962135 10.1016/j.msard.2021.102973

[20] Krause, I., Kern, S., Horntrich, A. & Ziemssen, T. Employment status in multiple sclerosis: impact of disease-specific and non-disease-specific factors. *Mult. Scler. J.* **19** (13), 1792–1799. 10.1177/1352458513485655 (2013). 10.1177/1352458513485655 23635910

[21] Passamonti, L. et al. Neurobiological mechanisms underlying emotional processing in relapsing-remitting multiple sclerosis. *Brain* **132** (12), 3380–3391. 10.1093/brain/awp095 (2009). 19420090 10.1093/brain/awp095

[22] Degraeve, B., Sequeira, H., Mecheri, H. & Lenne, B. Corpus callosum damage to account for cognitive, affective, and social-cognitive dysfunctions in multiple sclerosis: a model of callosal disconnection syndrome? *Mult. Scler. J.*10.1177/13524585221091067 (2022). 10.1177/13524585221091067 35475386

[23] Giazkoulidou, A., Messinis, L. & Nasios, G. Cognitive functions and social cognition in multiple sclerosis: an overview. *Hell J. Nucl. Med.* **22 Suppl**, 102–110 (2019). 30877728

[24] Ciampi, E. et al. Relationship between Social Cognition and traditional cognitive impairment in Progressive Multiple Sclerosis and possible implicated neuroanatomical regions. *Mult. Scler. Relat. Disord.* **20**, 122–128. 10.1016/j.msard.2018.01.013 (2018). 29414284 10.1016/j.msard.2018.01.013

[25] Batista, S. et al. Disconnection as a mechanism for social cognition impairment in multiple sclerosis. *Neurology* **89** (1), 38–45. 10 (2017). 28566550 10.1212/WNL.0000000000004060

[26] Golde, S. et al. Distinct functional connectivity signatures of impaired social cognition in multiple sclerosis. *Front. Neurol.* **11**, 507. 10.3389/fneur.2020.00507 (2020). 32670178 10.3389/fneur.2020.00507 PMC7330009

[27] Skowronski, J. J. & Carlston, D. E. Negativity and extremity biases in impression formation: a review of explanations. *Psychol. Bull.* **105** (1), 131–142. 10.1037/0033-2909.105.1.131 (1989).

[28] De Meo, E. et al. Identifying the distinct cognitive phenotypes in multiple sclerosis. *JAMA Neurol.* **78** (4), 414. 10.1001/jamaneurol.2020.4920 (2021). 33393981 10.1001/jamaneurol.2020.4920 PMC7783596

[29] Brochet, B. & Ruet, A. Cognitive impairment in multiple sclerosis with regards to disease duration and clinical phenotypes. *Front. Neurol.* **10**, 261. 10.3389/fneur.2019.00261 (2019). 30949122 10.3389/fneur.2019.00261 PMC6435517

[30] Leavitt, V. M., Tosto, G. & Riley, C. S. Cognitive phenotypes in multiple sclerosis. *J. Neurol.* **265** (3), 562–566. 10.1007/s00415-018-8747-5 (2018). 29356970 10.1007/s00415-018-8747-5

[31] Berneiser, J. et al. Impaired recognition of emotional facial expressions in patients with multiple sclerosis. *Mult. Scler. Relat. Disord.* **3** (4), 482–488. 10.1016/j.msard.2014.02.001 (2014). 25877060 10.1016/j.msard.2014.02.001

[32] Pottgen, J., Dziobek, I., Reh, S., Heesen, C. & Gold, S. M. Impaired social cognition in multiple sclerosis. *J. Neurol. Neurosurg. Psychiatry*. **84** (5), 523–528. 10.1136/jnnp-2012-304157 (2013). 23315621 10.1136/jnnp-2012-304157

[33] Henry, A. et al. Social cognition impairments in relapsing-remitting multiple sclerosis. *J. Int. Neuropsychol. Soc. JINS*. **17** (6), 1122–1131. 10 (2011). 22014035 10.1017/S1355617711001147

[34] Prochnow, D. et al. Alexithymia and impaired facial affect recognition in multiple sclerosis. *J. Neurol.* **258** (9), 1683–1688. 10.1007/s00415-011-6002-4 (2011). 21442462 10.1007/s00415-011-6002-4

[35] Chalah, M. A. & Ayache, S. S. Deficits in social cognition: an unveiled signature of multiple sclerosis. *J. Int. Neuropsychol. Soc. JINS*. **23** (3), 266–286. 10.1017/S1355617716001156 (2017). 28069095 10.1017/S1355617716001156

[36] Lohaus, T., Witt, J., Schürmeyer, A., Wolf, O. T. & Thoma, P. Fatigue and its relation to general cognition, social cognition and social activity in multiple sclerosis and stroke. *Cogn. Neuropsychiatry* **28** (3), 165–180. 10.1080/13546805.2023.2178399 (2023). 36782396 10.1080/13546805.2023.2178399

[37] Dulau, C. et al. Social cognition according to cognitive impairment in different clinical phenotypes of multiple sclerosis. *J. Neurol.* **264** (4), 740–748. 10.1007/s00415-017-8417-z (2017). 28220288 10.1007/s00415-017-8417-z

[38] Vetter, N. C., Leipold, K., Kliegel, M., Phillips, L. H. & Altgassen, M. Ongoing development of social cognition in adolescence. *Child. Neuropsychol.* **19** (6), 615–629. 10.1080/09297049.2012.718324 (2013). 22934659 10.1080/09297049.2012.718324

[39] Blakemore, S. J. & Choudhury, S. Development of the adolescent brain: implications for executive function and social cognition. *J. Child. Psychol. Psychiatry*. **47** (3–4), 296–312. 10.1111/j.1469-7610.2006.01611.x (2006). 16492261 10.1111/j.1469-7610.2006.01611.x

[40] Kurtzke, J. F. Rating neurologic impairment in multiple sclerosis: an expanded disability status scale (EDSS). *Neurology* **33** (11), 1444–1444. 10.1212/WNL.33.11.1444 (1983). 6685237 10.1212/wnl.33.11.1444

[41] Moher, D. et al. Preferred reporting items for systematic review and meta-analysis protocols (PRISMA-P) 2015 statement. *Syst. Rev.* **4** (1), 1. 10.1186/2046-4053-4-1 (2015). 25554246 10.1186/2046-4053-4-1 PMC4320440

[42] Shamseer, L. et al. Preferred reporting items for systematic review and meta-analysis protocols (PRISMA-P) 2015: elaboration and explanation. *BMJ* **349** (jan02 1), g7647–g7647. 10.1136/bmj.g7647 (2015). 10.1136/bmj.g7647 25555855

[43] Degraeve, B., Henry, A. & Lenne, B. Relationship between emotion recognition and cognition in multiple sclerosis: a meta-analysis protocol. *BMJ Neurol. Open.* **6** (1), e000471. 10.1136/bmjno-2023-000471 (2024). 38268751 10.1136/bmjno-2023-000471 PMC10806822

[44] Ghanavati, E., Salehinejad, M. A., Nejati, V. & Nitsche, M. A. Differential role of prefrontal, temporal and parietal cortices in verbal and figural fluency: implications for the supramodal contribution of executive functions. *Sci. Rep.* **9** (1), 3700. 10.1038/s41598-019-40273-7 (2019). 30842493 10.1038/s41598-019-40273-7 PMC6403289

[45] Henry, J. D. & Beatty, W. W. Verbal fluency deficits in multiple sclerosis. *Neuropsychologia* **44** (7), 1166–1174. 10.1016/j.neuropsychologia.2005.10.006 (2006). 16293271 10.1016/j.neuropsychologia.2005.10.006

[46] Delgado-Álvarez, A. et al. Cognitive processes underlying verbal fluency in multiple sclerosis. *Front. Neurol.* **11**, 629183. 10.3389/fneur.2020.629183 (2021). 33551984 10.3389/fneur.2020.629183 PMC7859643

[47] Rao, S. M., Leo, G. J. & Aubin-Faubert, P. On the nature of memory disturbance in multiple sclerosis. *J. Clin. Exp. Neuropsychol.* **11** (5), 699–712. 10.1080/01688638908400926 (1989). 10.1080/01688638908400926 2808659

[48] Krause, M. et al. Prefrontal function associated with impaired emotion recognition in patients with multiple sclerosis. *Behav. Brain Res.* **205** (1), 280–285. 10.1016/j.bbr.2009.08.009 (2009). 19686782 10.1016/j.bbr.2009.08.009

[49] Heberlein, A. S., Padon, A. A., Gillihan, S. J., Farah, M. J. & Fellows, L. K. Ventromedial frontal lobe plays a critical role in facial emotion recognition. *J. Cogn. Neurosci.* **20** (4), 721–733. 10.1162/jocn.2008.20049 (2008). 18052791 10.1162/jocn.2008.20049

[50] Tüdös, Z., Hok, P., Hrdina, L. & Hluštík, P. Modality effects in paced serial addition task: differential responses to auditory and visual stimuli. *Neuroscience* **272**, 10–20. 10.1016/j.neuroscience.2014.04.057 (2014). 24802163 10.1016/j.neuroscience.2014.04.057

[51] Haxby, J. V., Hoffman, E. A. & Gobbini, M. I. The distributed human neural system for face perception. *Trends Cogn. Sci.* **4** (6), 223–233. 10.1016/S1364-6613(00)01482-0 (2000). 10827445 10.1016/s1364-6613(00)01482-0

[52] Vuilleumier, P. & Pourtois, G. Distributed and interactive brain mechanisms during emotion face perception: evidence from functional neuroimaging. *Neuropsychologia* **45** (1), 174–194. 10.1016/j.neuropsychologia.2006.06.003 (2007). 16854439 10.1016/j.neuropsychologia.2006.06.003

[53] Pitcher, D., Walsh, V. & Duchaine, B. The role of the occipital face area in the cortical face perception network. *Exp. Brain Res.* **209** (4), 481–493. 10.1007/s00221-011-2579-1 (2011). 21318346 10.1007/s00221-011-2579-1

[54] Tsalouchidou, P. E. et al. Verbal memory depends on structural hippocampal subfield volume. *Front. Neurol.* **14**, 1209941. 10.3389/fneur.2023.1209941 (2023). 37900611 10.3389/fneur.2023.1209941 PMC10613087

[55] Mike, A. et al. Disconnection mechanism and regional cortical atrophy contribute to impaired processing of facial expressions and theory of mind in multiple sclerosis: a structural MRI study. *PLOS ONE*. **8** (12), e82422. 10.1371/journal.pone.0082422 (2013). 24349280 10.1371/journal.pone.0082422 PMC3862626

[56] Jehna, M. et al. Cognitively preserved MS patients demonstrate functional differences in processing neutral and emotional faces. *Brain Imaging Behav.* **5** (4), 241–251. 10.1007/s11682-011-9128-1 (2011). 21656213 10.1007/s11682-011-9128-1

[57] Pfaff, L. et al. Emotional disturbances in multiple sclerosis: a neuropsychological and fMRI study. *Cortex* **117**, 205–216. 10.1016/j.cortex.2019.02.017 (2019). 30991308 10.1016/j.cortex.2019.02.017

[58] Palermo, R. & Coltheart, M. Photographs of facial expression: accuracy, response times, and ratings of intensity. *Behav. Res. Methods Instrum. Comput.* **36** (4), 634–638. 10.3758/BF03206544 (2004). 15641409 10.3758/bf03206544

[59] Argaud, S., Vérin, M., Sauleau, P. & Grandjean, D. Facial emotion recognition in Parkinson’s disease: a review and new hypotheses. *Mov. Disord*. **33** (4), 554–567. 10.1002/mds.27305 (2018). 29473661 10.1002/mds.27305 PMC5900878

[60] Goodkind, M. S. et al. Emotion recognition in frontotemporal dementia and Alzheimer’s disease: a new film-based assessment. *Emotion* **15** (4), 416–427. 10.1037/a0039261 (2015). 26010574 10.1037/a0039261 PMC4551420

[61] Johnston, P. J., Devir, H. & Karayanidis, F. Facial emotion processing in schizophrenia: no evidence for a deficit specific to negative emotions in a differential deficit design. *Psychiatry Res.* **143** (1), 51–61. 10.1016/j.psychres.2005.08.006 (2006). 16725209 10.1016/j.psychres.2005.08.006

[62] Zikos, L. et al. Preservation of positive emotion recognition in patients with multiple sclerosis: Artefact related to methodological issues or real feature of the disorder? *J. Neuropsychol.*10.1111/jnp.70051 (2026). 10.1111/jnp.70051 PMC13552246 42104901

[63] Nummenmaa, L. & Calvo, M. G. Dissociation between recognition and detection advantage for facial expressions: a meta-analysis. *Emotion* **15** (2), 243–256. 10.1037/emo0000042 (2015). 25706834 10.1037/emo0000042

[64] Carstensen, L. L. Socioemotional selectivity theory: the role of perceived endings in human motivation. *The Gerontologist* **61** (8), 1188–1196. 10.1093/geront/gnab116 (2021). 10.1093/geront/gnab116 PMC8599276 34718558

[65] Kellough, J. L. & Knight, B. G. Positivity effects in older adults’ perception of facial emotion: the role of future time perspective. *J. Gerontol. B Psychol. Sci. Soc. Sci.* **67B** (2), 150–158. 10.1093/geronb/gbr079 (2012). 10.1093/geronb/gbr079 21798858

[66] Reed, A. E., Chan, L. & Mikels, J. A. Meta-analysis of the age-related positivity effect: age differences in preferences for positive over negative information. *Psychol. Aging*. **29** (1), 1–15. 10.1037/a0035194 (2014). 24660792 10.1037/a0035194

[67] Dow, K. H., Ferrell, B. R., Haberman, M. R. & Eaton, L. The meaning of quality of life in cancer survivorship. *Oncol. Nurs. Forum*. **26** (3), 519–528 (1999). 10214594

[68] Horgan, O., Holcombe, C. & Salmon, P. Experiencing positive change after a diagnosis of breast cancer: a grounded theory analysis. *Psychooncology* **20** (10), 1116–1125. 10.1002/pon.1825 (2011). 20734340 10.1002/pon.1825

[69] Milam, J. E. Posttraumatic growth among HIV/AIDS patients. *J. Appl. Soc. Psychol.* **34** (11), 2353–2376. 10.1111/j.1559-1816.2004.tb01981.x (2004).

[70] Petrie, K. J., Buick, D. L., Weinman, J. & Booth, R. J. Positive effects of illness reported by myocardial infarction and breast cancer patients. *J. Psychosom. Res.* **47** (6), 537–543. 10.1016/S0022-3999(99)00054-9 (1999). 10661601 10.1016/s0022-3999(99)00054-9

[71] Schroevers, M. J., Kraaij, V. & Garnefski, N. Cancer patients’ experience of positive and negative changes due to the illness: relationships with psychological well-being, coping, and goal reengagement. *Psychooncology* **20** (2), 165–172. 10.1002/pon.1718 (2011). 20217657 10.1002/pon.1718

[72] Zikos, L. et al. Distinguishing the role of positivity bias, cognitive impairment and emotional reactivity in the deontological preference in multiple sclerosis during moral dilemmas: a social cognition study protocol. *Front. Psychol.* **15**, 1404876. 10.3389/fpsyg.2024.1404876 (2024). 39091703 10.3389/fpsyg.2024.1404876 PMC11291456

[73] Noordewier, M. K. & Breugelmans, S. M. On the valence of surprise. *Cogn. Emot.* **27** (7), 1326–1334. 10.1080/02699931.2013.777660 (2013). 23560688 10.1080/02699931.2013.777660

[74] Degraeve, B., Lenne, B., Norberciak, L., Massot, C. & Donze, C. A comparative analysis of depression screening tools in multiple sclerosis: implications for diagnosis and prevalence. *Mult Scler. Relat. Disord*. **93**, 106220. 10.1016/j.msard.2024.106220 (2025). 39709698 10.1016/j.msard.2024.106220

[75] Isernia, S. et al. Resting-state functional brain connectivity for human mentalizing: biobehavioral mechanisms of theory of mind in multiple sclerosis. *Soc. Cogn. Affect. Neurosci.* **17** (6), 579–589. 10.1093/scan/nsab120 (2022). 34748015 10.1093/scan/nsab120 PMC9164209

[76] Gil-González, I., Martín-Rodríguez, A., Conrad, R. & Pérez-San-Gregorio, M. Á. Quality of life in adults with multiple sclerosis: a systematic review. *BMJ Open.* **10** (11), e041249. 10.1136/bmjopen-2020-041249 (2020). 33257490 10.1136/bmjopen-2020-041249 PMC7705559

