# Supplementary material for: Association between emotion recognition and cognitive functioning in multiple sclerosis: a multilevel correlational meta-analysis
Source: Sci Rep. 2026 May 29;16:26209. doi: 10.1038/s41598-026-52969-8 (PMC13493921; doi:10.1038/s41598-026-52969-8)
Supplement: Supplementary file 1 [file 41598_2026_52969_MOESM1_ESM.docx]

[Section S1, Extracted variables and coding procedures	2](#_Toc229048220)

[Table S1. Definition of all variables for which data were sought.	2](#_Toc229048221)

[Section S2, Overview of Cognitive Measures and Domain Classification	3](#_Toc229048222)

[Table S2. Classification of cognitive measures by domain and subdomain	3](#_Toc229048223)

[Section S3, Statistical models and estimation procedures	4](#_Toc229048224)

[Section S4, Quality Assessment of Included Studies	6](#_Toc229048225)

[Table S3. Methodological quality ratings based on sample size, measure validation, and control of confounders	7](#_Toc229048226)

[Section S5, Overall Publication bias	11](#_Toc229048227)

[Table S4. Egger’s Regression Test Results by Emotion–Domain Combination (Overall)	11](#_Toc229048228)

[Figure S1. Funnel plots by Emotion–Domain Combination (Overall)	11](#_Toc229048229)

[Figure S2. Non-aggregated plots showing the correlations between emotional recognition and executive functions, grouped by cognitive subdomain, for each emotion.	12](#_Toc229048230)

[Section S6, Publication Bias Within Executive Subdomains	13](#_Toc229048231)

[Figure S3. Funnel plots by Emotion–Executive subdomains Combination	13](#_Toc229048232)

[Table S5. Egger’s Regression Test Results by Emotion– Executive subdomains Combination	13](#_Toc229048233)

[Section S7, Publication Bias Within Episodic Memory Subdomains	14](#_Toc229048234)

[Table S6. Egger’s Regression Test Results by Emotion–Episodic Memory subdomains Combination	14](#_Toc229048235)

[Figure S4. Funnel plots by Emotion– Episodic Memory subdomains Combination	14](#_Toc229048236)

[Section S8, Publication Bias Within IPS Subdomains	15](#_Toc229048237)

[Table S7. Egger’s Regression Test Results by Emotion–IPS subdomains Combination	15](#_Toc229048238)

[Figure S5. Funnel plots by Emotion–IPS subdomains Combination	15](#_Toc229048239)

[Table S8. Meta-Regression Analyses of Clinical and Demographic Moderators	15](#_Toc229048240)

[Section S9, Forest Plots	16](#_Toc229048241)

[Figure S6. Forest plots showing the correlations between emotional recognition and executive functions, grouped by cognitive subdomain, for each emotion.	16](#_Toc229048242)

[Figure S7. Forest plots showing the correlations between emotional recognition and Episodic Memory, grouped by cognitive subdomain, for each emotion.	18](#_Toc229048243)

[Figure S8. Forest plots showing the correlations between emotional recognition and IPS, grouped by cognitive subdomain, for each emotion.	20](#_Toc229048244)

[Section S10, PRISMA 2020 Checklist	22](#_Toc229048245)

Section S1, Extracted variables and coding procedures

All variables are listed and defined in Table S1 below.

## Table S1. Definition of all variables for which data were sought.

| **Variable** | **Definition** |
| --- | --- |
| **Population** | The population of interest was (adult) patients diagnosed with MS. |
| **Primary outcomes measures** | Primary outcomes consist of correlation coefficients between emotion recognition and cognitive performance in patients.  An emotion recognition task is defined as one requiring participants to discriminate the emotion being expressed by static or dynamic faces depicting basic (anger, disgust, fear, happiness, surprise, and sadness) or complex (e.g., pride, shame…) emotions. |
| **Subgroup analyses** | **Sub-scores of the emotion recognition task:** We identified 7 scores of interests: a total score and six basic emotion subscores.  **Cognitive Domains assessed by the cognitive measure:** We identified 3 cognitive domains of interests: Information Processing speed, Executive functions, Episodic memory. |
| **Additional outcomes (Moderators)** | We identified potential moderators including demographic and clinical variables (including age, disease duration, EDSS score, severity of depression, severity of anxiety, fatigue, metacognition and alexithymia). |

*Note*. “Total” emotion recognition scores were extracted as reported in the original studies. In most cases, these scores reflected performance on basic emotions, although one included study (Yap et al., 2022) used a broader set of emotional concepts.

# Section S2, Overview of Cognitive Measures and Domain Classification

The classification of neuropsychological tests into cognitive domains and subdomains was informed by previous large-scale meta-analyses and neuropsychological frameworks (e.g., [41], see Table S2). Minor adaptations were made when necessary to align with the specific goals of the present synthesis.

Specifically, the *Paced Auditory Serial Addition Test* (PASAT[42]) was treated as a distinct subdomain of processing speed, given its frequent conceptualization in MS research as a measure of IPS, despite its additional working memory demands. It was distinguished from SDMT-type tasks to allow for more precise domain-level analysis, given that SDMT is almost always included when PASAT is administered in MS studies. In the same vein, verbal fluency tasks were categorized separately from cognitive flexibility measures, following the approach of Fett et al. (2011)[41], but also because both types of tasks are often administered concurrently in MS studies (e.g., verbal fluency in addition to the Trail Making Test). This distinction is also clinically relevant, as verbal fluency is considered a sensitive indicator of executive dysfunction in MS population [43].

## Table S2. Classification of cognitive measures by domain and subdomain

| **Cognitive Domain** | **Subdomain** | **Tests / Measures** |
| --- | --- | --- |
| **Episodic Memory** | Verbal Episodic Memory | California Verbal Learning Test (CVLT, CVLT-II); RL/RI-16; Screening Examination for Cognitive Impairment (SEFCI, verbal memory subtest); Selective Reminding Test (SRT) |
|  | Visual Episodic Memory | 10/36 Spatial Recall Test; 7/24 Spatial Recall Test, SPART; Brief Visuospatial Memory Test – Revised (BVMT-R) |
| **Executive Functions** | Abstraction, Reasoning | Brixton Spatial Anticipation Test; Cognitive Estimation Test (CET); WAIS Similarities subtest; Wisconsin Card Sorting Test (WCST) |
|  | Attention | Brief Test of Attention (BTA); Divided Attention TAP (number of omissions); Db Task |
|  | Flexibility | Brixton; Cross Tapping (errors); TAP subtest; Trail Making Test (TMT) |
|  | Fluency | Phonemic Fluency (e.g., words beginning with P or R); Semantic Fluency; Verbal Fluency tests (e.g., WLG, COWAT) |
|  | Inhibition | Color-Word Interference Test (e.g., Stroop, D-KEFS CWIT); Go/No-Go (G/NG); TAP subtest. |
|  | Planning | Tower of London |
|  | Working Memory | Corsi Block-Tapping Task; Digit Span. |
| **Information Processing Speed** | SDMT and its variants | Symbol Digit Modalities Test (SDMT); Digit Symbol Coding (WAIS) |
|  | PASAT | Paced Auditory Serial Addition Test (PASAT, PASAT 3); PASAT variants including backward/forward spans |

*Note*. The term “subdomain” is used here to facilitate interpretation, although some categories (e.g., PASAT vs. SDMT) refer to distinct assessments rather than theoretically defined subcomponents. This distinction was retained to reflect their differential cognitive demands and frequent co-administration in MS research.

# Section S3, Statistical models and estimation procedures

Combined effects were computed in R using multilevel random-effects models implemented in the metafor package [60]. This approach accounts for the dependency structure of the data when multiple effect sizes are extracted from the same study.

The mean effect size (*r*) and 95% confidence intervals (CI) were used to assess the overall association between emotion recognition and cognition in patients. Effect sizes are weighted by the inverse of their sampling variance.

For missing data, we contacted the first or corresponding authors of the included studies via email to obtain relevant information not reported in the original articles. All retrieved correlations were transformed using Fisher’s *z* transformation prior to synthesis to reduce bias associated with combining *r* coefficient effect sizes [61]. Correlation signs were adjusted such that higher cognitive scores reflect better performance. For time-based tasks (e.g., Trail Making Test, Stroop, dual tasks costs….), raw correlation signs were reversed for interpretability.

**S3.1 Assessment of heterogeneity**

We assessed heterogeneity using several complementary indices. The *Q* statistic was computed to test for residual heterogeneity across effect sizes. We also computed the *I*² index, which reflects the percentage of variance attributable to real differences between studies rather than sampling error [62], with *I*^2^  < 50 indicating low heterogeneity [63]. In addition, we estimated the between-study variance ($\tau$²) using restricted maximum likelihood (REML), a recommended method in multilevel random-effects meta-analysis [64].

**S3.2 Assessment of publication bias**

We used funnel plots to detect publication bias. If the analysis included ≥ 10 studies, a test for funnel plot asymmetry using Egger method was conducted [65].

**S3.3 Subgroup and Meta-regression analyses**

We performed separate subgroup analyses (as listed in Table 1) to investigate associations between emotion recognition (7 scores of interests: total score and by 6-basic emotions sub-scores) and 3 cognitive domains. Additionally, for each subgroup of analyses, we conducted random-effects multilevel meta-regression analyses to investigate moderating effects of additional identified variables (as listed in Table 1). Specifically, meta-regression analyses were conducted to investigate whether demographic and clinical variables (including age, disease duration, EDSS score, severity of depression, severity of anxiety, fatigue, and alexithymia) are the cause for heterogeneity in any combined subgroup associations. We investigated mean age, disease duration, EDSS scores as continuous moderators using meta-regression. Regarding severity of depression, anxiety, fatigue, and alexithymia, we classified studies according to accepted cut-off scores of the used rating scales (no symptoms = 0, mild symptoms = 1, moderate symptoms = 2, severe symptoms = 3). Only combinations including at least 3 studies were considered for meta-regression analyses. All models were estimated using restricted maximum likelihood (REML).

# Section S4, Quality Assessment of Included Studies

In line with PRISMA guidelines, we conducted a quality assessment of the included studies using four predefined criteria: (1) sample size of the MS group, (2) use of validated cognitive and emotion recognition measures, (3) consideration of confounding variables (e.g., depression, fatigue, disability), and (4) clarity of data reporting. Each study received a qualitative global rating (“1-Low”, “2-Moderate”, or “3-High”) based on the number of criteria met.

Quality ratings were based on four criteria: (1) sample size (score = 3 if ≥ 30 MS patients; 2 if between 20-29; 1 if < 20), (2) validated cognitive measures (3 = established neuropsychological tools; 2 = limited or unclear validity; 1 = non-standard); (3) validated emotion recognition measures (3 = standardized and validated emotion recognition batteries; 2 = limited or unclear validity; 1 = non-standard); (4) consideration of confounding variables (3 = controlled statistically or via inclusion criteria; 2 = mentioned but not controlled; 1 = not addressed), and (5) clear reporting of correlation data.

We did not include clarity of correlation reporting as a formal quality criterion, as most included studies did not set emotion–cognition associations as their primary objective. However, we noted that in a number of cases, correlations had to be extracted from Tables, Figures or supplementary materials, or requested from authors.

Consideration of confounding variables was defined as either (a) the statistical control of one or more clinical variables known to influence cognition or emotion recognition (e.g., depression, anxiety, fatigue, alexithymia) through covariate adjustment or partial correlations, or (b) sample-level control, such as the exclusion of participants with moderate-to-severe symptoms or reporting of subgroup comparisons. Studies that did not report or address any of these factors were rated as not considering confounders.

Studies were rated as “Low”, “Moderate”, or “High” quality based on their cumulative score across the four criteria (maximum = 12). A score of 10-12 indicated high quality, reflecting either studies that met at least three criteria at the highest level (score = 3), or studies with two high and two moderate ratings (i.e., two scores = 3 and two scores = 2, without major methodological weaknesses). A score of 7-9 indicated moderate quality, and ≤ 6 low quality.

The study quality assessment results are shown in Table S3. The mean score was 11.60 (*SD* = 0.66), and all included studies were awarded ≥ 10 points and considered to be of high quality.

## Table S3. Methodological quality ratings based on sample size, measure validation, and control of confounders

| **Study (Author, Year)** | **C1. Sample Size (MS)** | | | | **C2. Validated Cognitive Measures** | | **C3. Validated Emotion Recognition Measures** | | | **C4. Confounders Addressed** | | | **C5. Correlation data accessible** | | **Overall Quality** | | |
| --- | --- | --- | --- | --- | --- | --- | --- | --- | --- | --- | --- | --- | --- | --- | --- | --- | --- |
| Dulau et al., 2017[37] | 60 | 30 RRMS, 15 PPMS, 15 SPMS | 3 | Established neuropsychological tools | | 3 | Bordeaux Social Cognition Evaluation Protocol (PECS-B, Etchepare et al., 2014) | 3 | EDSS, BDI, STAI, BAVQ | | 3 | Extracted from Tables, Figures, or supplementary materials (not fully available) | | 12 | | High |  |
| Aktuna et al., 2024[44] | 120 | RRMS | 3 | Established neuropsychological tools | | 3 | Facial Emotion Identification Test (FEIT, Erol et al., 2009) | 3 | EDSS, BDI | | 3 | Extracted from Tables, Figures, or supplementary materials (not fully available) | | 12 | | High |  |
| Berneiser et al. 2014[31] | 61 | 47 RR, 11 SP, 3 PP | 3 | Established neuropsychological tools | | 3 | Florida Affect Battery (FAB, Bower, Blonder, & Heilman, 1999) | 3 | EDSS, BDI, MS-FS | | 3 | Extracted from Tables, Figures, or supplementary materials (not fully available) | | 12 | | High |  |
| Cecchetto et al., 2014[11] | 30 | RRMS | 3 | Established neuropsychological tools | | 3 | NimStim stimuli set (Tottenham et al., 2009) | 3 | EDSS, BDI-FS, TAS-20, Fatigue Severity Scale | | 3 | Requested from authors | | 12 | | High |  |
| Delgado-Álvarez et al., 2021[45] | 39 | RRMS | 3 | Established neuropsychological tools | | 3 | Faces–Emotion Labeling task (Ebner, Riediger, & Lindenberge, 2010) | 3 | EDSS, BDI-II | | 3 | Requested from authors | | 12 | | High |  |
| Ehrlé et al., 2020[46] | 21 | RRMS | 2 | Established neuropsychological tools | | 3 | French computerized battery (Batterie de Cognition Sociale, BCS, Ehrlé, 2015) | 3 | EDSS | | 3 | Requested from authors | | 11 | | High |  |
| Ehrlé et al., 2020[47] | 45 | RRMS | 3 | Established neuropsychological tools | | 3 | French computerized battery (Batterie de Cognition Sociale, BCS, Ehrlé, 2015) | 3 | EDSS | | 3 | Requested from authors | | 12 | | High |  |
| Genova et al., 2020[48] | 17 | 9 PPMS, 4 SPMS, 2 PPMS | 1 | Established neuropsychological tools | | 3 | TASIT-S, Emotion Evaluation Task (EET, Honan et al., 2016) | 3 | EDSS not reported | | 3 | Extracted from Tables, Figures, or supplementary materials (not fully available) | | 10 | | High |  |
| Henry et al., 2009[12] | 27 | RRMS | 2 | Established neuropsychological tools | | 3 | Ekman Faces tasks (Ekman & Friesen, 1976) | 3 | EDSS, GDS | | 3 | Extracted from Tables, Figures, or supplementary materials (not fully available) | | 11 | | High |  |
| Henry et al., 2011[33] | 64 | RRMS | 3 | Established neuropsychological tools | | 3 | Ekman Faces tasks (Ekman & Friesen, 1976) | 3 | EDSS, BDI-FS, EMIF-SEP | | 3 | Requested from authors | | 12 | | High |  |
| Henry et al., 2017[10] | 62 | 31 RRMS, 16 PPMS, 15 SPMS | 3 | Established neuropsychological tools | | 3 | Ekman Faces tasks (Ekman & Friesen, 1976) | 3 | EDSS, HADS, BVAQ-B, EMIF-SEP | | 3 | Requested from authors | | 12 | | High |  |
| Henry et al., 2022[49] | 106 | 16 PPSM, 15 SPMS, 75 RRMS | 3 | Established neuropsychological tools | | 3 | Ekman Faces tasks (Ekman & Friesen, 1976) | 3 | EDSS, HADS, EMIF-SEP | | 3 | Requested from authors | | 12 | | High |  |
| Jehna et al., 2011[50] | 15 | RRMS | 1 | Established neuropsychological tools | | 3 | Karolinska Directed Emotional Faces database (KDEF, Lundqvist et al. 1998) | 3 | EDSS, BDI | | 3 | Requested from authors | | 10 | | High |  |
| Lenne et al., 2015[13] | 50 | RRMS | 3 | Established neuropsychological tools | | 3 | Ekman Faces tasks (Ekman & Friesen, 1976) | 3 | EDSS, BDI, STAI, TAS-20, FIS | | 3 | Requested from authors | | 12 | | High |  |
| Mestdagh et al., 2021[51] | 32 | 31 RRMS, 1 PPMS | 3 | Established neuropsychological tools | | 3 | Ekman Faces tasks (Ekman & Friesen, 1976) | 3 | EDSS, BDI-FS, no diesase duration | | 3 | Extracted from Tables, Figures, or supplementary materials (not fully available) | | 12 | | High |  |
| Montembeault et al., 2022[52] | 29 | 20 RRMS, 7 SPMS, 2 PPMS | 2 | Established neuropsychological tools | | 3 | NimStim stimuli set (Tottenham et al., 2009) + Montreal Affective Voices stimuli set (Belin et al., 2008) | 3 | EDSS, BDI-FS, MFIS | | 3 | Requested from authors | | 11 | | High |  |
| Pfaff et al., 2021[53] | 25 | RRMS | 2 | Established neuropsychological tools | | 3 | Florida Affect Battery (FAB, Bower, Blonder, & Heilman, 1999) | 3 | EDSS, EHD, MFIS | | 3 | Requested from authors | | 11 | | High |  |
| Phillips et al., 2011[54] | 32 | 27 RRMS, 2 PPMS, 3 SPMS | 3 | Established neuropsychological tools | | 3 | Emotion recognition task adapted from Sullivan and Ruffman (2004) | 3 | Disease Steps, HADS | | 3 | Requested from authors | | 12 | | High |  |
| Radlak et al., 2021[55] | 53 | 29 RRMS, 21 SPMS, 3 PPMS | 3 | Established neuropsychological tools | | 3 | Ekman Faces tasks (Ekman & Friesen, 1976) + Florida Affect Battery (FAB, Bower, Blonder, & Heilman, 1999) | 3 | EDSS | | 3 | Extracted from Tables, Figures, or supplementary materials (not fully available) | | 12 | | High |  |
| Realmuto et al., 2019[56] | 45 | RRMS | 3 | Established neuropsychological tools | | 3 | Ekman Faces tasks (Ekman & Friesen, 1976) | 3 | EDSS, HADS (but no subscore available), FSS | | 3 | Requested from authors | | 12 | | High |  |
| Yap et al., 2022[57] | 45 | 21 RRMS, 19 SPMS, 5 PPMS | 3 | Established neuropsychological tools | | 3 | Cambridge Mindreading FAce-Voice Battery | 3 | EDSS, HADS, MFIS | | 3 | Extracted from Tables, Figures, or supplementary materials (not fully available) | | 12 | | High |  |
| Ziccardi et al., 2021[58] | 42 | RRMS | 3 | Established neuropsychological tools | | 3 | Karolinska Directed Emotional Faces database (KDEF, Lundqvist et al. 1998) | 3 | EDSS, DASS-21, | | 3 | Requested from authors | | 12 | | High |  |
| Zikos et al., in preparation[59] | 50 | RRMS | 3 | Established neuropsychological tools | | 3 | EU-Emotion Stimulus Set (O’Reilly et al., 2016) | 3 | EDSS, BDI-FS, STAI, TAS-20 | | 3 | Requested from authors | | 12 | | High |  |

*Note*. RRMS = Relapsing-Remitting Multiple Sclerosis; SPMS = Secondary Progressive Multiple Sclerosis; PPMS = Primary Progressive Multiple Sclerosis; EDSS = Expanded Disability Status Scale; MFIS = Modified Fatigue Impact Scale; BDI = Beck Depression Inventory, BDI-FS = Beck Depression Inventory - Fast Screen; GDS = Geriatric Depression Scale; HADS = Hospital Anxiety and Depression; DASS= Depression Anxiety and Stress Scale; STAI = State-Trait Anxiety Inventory; TAS-20 = Toronto Alexithymia Scale; BVAQ-B = Bermond–Vorst Alexithymia Questionnaire; EMIF-SEP = Echelle modifiée d’Impact de la Fatigue – Sclérose en Plaques; FSS = Fatigue Severity Scale, EHD : Echelle d’Humeur Dépressive.

# Section S5, Overall Publication bias

To assess potential small-study effects, Egger’s test was applied to each Emotion--Domain combination. The test examines whether the size of reported effects correlates with their standard error, which may indicate publication bias.

Egger’s regression test identified significant asymmetry in five Emotion--Domain combinations: Happiness across all three cognitive domains (executive function, episodic memory, and IPS), Neutral--Executive, and Total--Episodic Memory. These combinations all had sufficiently large sample sizes (*k* ranging from 18 to 71), supporting the reliability of the test and suggesting potential publication bias.

One additional combination (Surprise--Episodic Memory) also yielded significant Egger’s tests, but its low sample size (*k* = 10) warrants caution in interpretation. For all other combinations, Egger’s test was non-significant (*p* > .05), providing no indication of small-study effects.

Visual inspection of funnel plots provided mixed evidence regarding the presence of small-study effects. For Neutral--Executive and Total--Episodic Memory, although Egger’s tests were statistically significant, the corresponding funnel plots appeared rather symmetrical, with data points distributed evenly around the pooled estimate. Despite the significant Egger’s test, the visual pattern did not clearly suggest publication bias.

For Happiness--Episodic Memory and Happiness--IPS, the distribution was moderately asymmetric, with a concentration of small studies reporting negative or null associations, consistent with the significant Egger’s test. For Happiness--Executive, the plot showed a denser distribution of studies reporting positive effects, but without striking asymmetry.

Funnel plots for all combinations are provided in Supplementary Figure S1. Detailed results of Egger’s tests for all Emotion–Domain combinations are presented in Supplementary Table S4.

## Table S4. Egger’s Regression Test Results by Emotion–Domain Combination (Overall)

<https://doi.org/10.17605/OSF.IO/3VNA4>

## Figure S1. Funnel plots by Emotion–Domain Combination (Overall)

<https://doi.org/10.17605/OSF.IO/3VNA4>

## Figure S2. Non-aggregated plots showing the correlations between emotional recognition and executive functions, grouped by cognitive subdomain, for each emotion.

<https://doi.org/10.17605/OSF.IO/3VNA4>

# Section S6, Publication Bias Within Executive Subdomains

To assess small-study effects within executive subdomains, Egger’s regression tests were conducted for each Emotion--Subdomain combination. Egger’s regression test identified significant asymmetry in three Emotion--Domain combinations: Happiness across two executive subdomains (Fluency and Working Memory) and Total--Fluency. These combinations all had sufficiently large sample sizes (*k* ranging from 13 to 33), supporting the reliability of the test and suggesting potential publication bias. Two additional combinations (Fear--Inhibition and Neutral--Abstraction) also yielded significant Egger’s tests, but its low sample size (with respectively *k* = 10 and *k* = 3) warrants caution in interpretation.

Visual inspection of the funnel plot revealed a relatively revealed moderate asymmetry, particularly for the Happiness--Fluency and Total--Fluency combinations, with smaller studies tending to report more extreme effect sizes, consistent with the Egger’s test results. Funnel plots for all combinations are provided in Supplementary Figure S3. Detailed results of Egger’s tests for all Emotion–Executive Subdomain combinations are presented in Supplementary Table S5.

## Figure S3. Funnel plots by Emotion–Executive subdomains Combination

<https://doi.org/10.17605/OSF.IO/3VNA4>

## Table S5. Egger’s Regression Test Results by Emotion– Executive subdomains Combination

<https://doi.org/10.17605/OSF.IO/3VNA4>

# Section S7, Publication Bias Within Episodic Memory Subdomains

To assess the presence of small-study effects in the episodic memory domain, Egger’s regression tests were conducted for each Emotion--Subdomain combination. We identified significant asymmetry in five Emotion--Memory combinations: Happiness--Verbal, Surprise--Verbal, Sadness--Visual, Anger--Visual, and Total--Visual (*p*s < .05). However, these combinations involved small samples (*k* between 5 and 10), warranting cautious interpretation.

Visual inspection of funnel plots provided mixed support for the significant Egger’s test results. For Happiness--Verbal, Anger--Visual, and Sadness--Visual, plots appeared relatively symmetrical, with a few outliers potentially driving the statistical asymmetry. In contrast, Surprise--Verbal showed a more irregular distribution, though the small number of studies (*k* = 5) limits interpretability. Total--Visual displayed slight asymmetry, driven by one extreme effect. Overall, visual evidence for publication bias remained weak.

Funnel plots for all combinations are provided in Supplementary Figure S4. Detailed results of Egger’s tests for all Emotion–Episodic Memory Subdomain combinations are presented in Supplementary Table S6.

## Table S6. Egger’s Regression Test Results by Emotion–Episodic Memory subdomains Combination

<https://doi.org/10.17605/OSF.IO/3VNA4>

## Figure S4. Funnel plots by Emotion– Episodic Memory subdomains Combination

<https://doi.org/10.17605/OSF.IO/3VNA4>

# Section S8, Publication Bias Within IPS Subdomains

To assess small-study effects within IPS subdomains, Egger’s regression tests were conducted for each Emotion–Subdomain combination. Only one trend-level asymmetry emerged for the combination of Happiness--SDMT (*k* = 12, *p* = .07), suggesting potential small-study effects or publication bias. Given the relatively modest number of included samples, this result should be interpreted cautiously. No other IPS subdomain showed signs of asymmetry, supporting the overall robustness of the IPS function findings.

However, visual inspection of the funnel plot (see Supplementary Figure S5) revealed a relatively symmetrical distribution of studies around the pooled estimate, with no clear indication of systematic small-study bias. Funnel plots for all combinations are provided in Supplementary Figure S5. Detailed results of Egger’s tests for all Emotion–IPS Subdomain combinations are presented in Supplementary Table S7.

## Table S7. Egger’s Regression Test Results by Emotion–IPS subdomains Combination

<https://doi.org/10.17605/OSF.IO/3VNA4>

## Figure S5. Funnel plots by Emotion–IPS subdomains Combination

<https://doi.org/10.17605/OSF.IO/3VNA4>

## Table S8. Meta-Regression Analyses of Clinical and Demographic Moderators

<https://doi.org/10.17605/OSF.IO/3VNA4>

# Section S9, Forest Plots

## Figure S6. Forest plots showing the correlations between emotional recognition and executive functions, grouped by cognitive subdomain, for each emotion.

*Note*. Each panel (A–H) corresponds to a specific emotion: A = Anger, B = Total Score, C = Fear, D = Happiness, E = Surprise, F = Sadness, G = Neutral, H = Disgust. Correlations (Fisher’s *z*) are grouped by executive function subdomain: Attention, Working Memory, Fluency, Flexibility, Inhibition, Planning, and Abstraction. Multilevel meta-analytic models were originally used to account for dependence between effect sizes within studies. For clarity, forest plots present individual estimates after model aggregation across studies. High Quality Forest plots (aggregated and non-aggregated) can be downloaded on the *Open Science Framework* (see Section 5. Pre-registration and data availability).

## Figure S7. Forest plots showing the correlations between emotional recognition and Episodic Memory, grouped by cognitive subdomain, for each emotion.

*Note*. Each panel (A–H) corresponds to a specific emotion: A = Anger, B = Total Score, C = Surprise, D = Sadness, E = Neutral, F = Happiness, G = Fear, H = Disgust. Correlations (Fisher’s *z*) are grouped by Episodic Memory subdomain: Verbal and Visual. Multilevel meta-analytic models are used to account for dependence between effect sizes within studies. High Quality Forest plots can be downloaded on the *Open Science Framework* (see Section 5. Pre-registration and data availability).

## Figure S8. Forest plots showing the correlations between emotional recognition and IPS, grouped by cognitive subdomain, for each emotion.

*Note*. Each panel (A–H) corresponds to a specific emotion: A = Anger, B = Total Score, C = Surprise, D = Sadness, E = Neutral, F = Happiness, G = Fear, H = Disgust. Correlations (Fisher’s *z*) are grouped by IPS subdomain: SDMT and PASAT. Multilevel meta-analytic models are used to account for dependence between effect sizes within studies. High Quality Forest plots can be downloaded on the *Open Science Framework* (see Section 5. Pre-registration and data availability).

# Section S10, PRISMA 2020 Checklist

| **Section and Topic** | **Item #** | **Checklist item** | **Location where item is reported** |
| --- | --- | --- | --- |
| **TITLE** | | |  |
| Title | 1 | Identify the report as a systematic review. | Title |
| **ABSTRACT** | | |  |
| Abstract | 2 | See the PRISMA 2020 for Abstracts checklist. | Abstract |
| **INTRODUCTION** | | |  |
| Rationale | 3 | Describe the rationale for the review in the context of existing knowledge. | *Section 1.1 Background* |
| Objectives | 4 | Provide an explicit statement of the objective(s) or question(s) the review addresses. | *Section 1.2 Objectives* |
| **METHODS** | | |  |
| Eligibility criteria | 5 | Specify the inclusion and exclusion criteria for the review and how studies were grouped for the syntheses. | *Section 2.1 Eligibility criteria (2.1.1 and 2.1.2)* |
| Information sources | 6 | Specify all databases, registers, websites, organisations, reference lists and other sources searched or consulted to identify studies. Specify the date when each source was last searched or consulted. | *Section 2.2.1 Data sources* |
| Search strategy | 7 | Present the full search strategies for all databases, registers and websites, including any filters and limits used. | *Section 2.2.2 Search strategy + Supplementary Material S1* |
| Selection process | 8 | Specify the methods used to decide whether a study met the inclusion criteria of the review, including how many reviewers screened each record and each report retrieved, whether they worked independently, and if applicable, details of automation tools used in the process. | *Section 2.2.2 Search strategy (study selection process)* |
| Data collection process | 9 | Specify the methods used to collect data from reports, including how many reviewers collected data from each report, whether they worked independently, any processes for obtaining or confirming data from study investigators, and if applicable, details of automation tools used in the process. | *Section 2.3 Data collection and analysis* |
| Data items | 10a | List and define all outcomes for which data were sought. Specify whether all results that were compatible with each outcome domain in each study were sought (e.g. for all measures, time points, analyses), and if not, the methods used to decide which results to collect. | *Section 2.3 Data collection and analysis (outcomes extracted)* |
|  | 10b | List and define all other variables for which data were sought (e.g. participant and intervention characteristics, funding sources). Describe any assumptions made about any missing or unclear information. | *Section 2.3 Data collection and analysis (sample and clinical variables)* |
| Study risk of bias assessment | 11 | Specify the methods used to assess risk of bias in the included studies, including details of the tool(s) used, how many reviewers assessed each study and whether they worked independently, and if applicable, details of automation tools used in the process. | *Supplementary Material S4 – Quality assessment of included studies* |
| Effect measures | 12 | Specify for each outcome the effect measure(s) (e.g. risk ratio, mean difference) used in the synthesis or presentation of results. | *Section 5.1 Summary of overall associations* |
| Synthesis methods | 13a | Describe the processes used to decide which studies were eligible for each synthesis (e.g. tabulating the study intervention characteristics and comparing against the planned groups for each synthesis (item #5)). | *Section 2.3 and Figure 1 (PRISMA flow)* |
|  | 13b | Describe any methods required to prepare the data for presentation or synthesis, such as handling of missing summary statistics, or data conversions. | *Supplementary S3 – data preparation and transformations* |
|  | 13c | Describe any methods used to tabulate or visually display results of individual studies and syntheses. | Tables 2–5 and forest plots (Supplementary S9) |
|  | 13d | Describe any methods used to synthesize results and provide a rationale for the choice(s). If meta-analysis was performed, describe the model(s), method(s) to identify the presence and extent of statistical heterogeneity, and software package(s) used. | *Section 2.3 / Statistical models (Supplementary S3)* |
|  | 13e | Describe any methods used to explore possible causes of heterogeneity among study results (e.g. subgroup analysis, meta-regression). | *Sections 5.2 and 5.3 (subgroup analyses and meta-regressions)* |
|  | 13f | Describe any sensitivity analyses conducted to assess robustness of the synthesized results. | *Supplementary S6–S8 (analyses de sensibilité)* |
| Reporting bias assessment | 14 | Describe any methods used to assess risk of bias due to missing results in a synthesis (arising from reporting biases). | *Sections 5.1.2 et Supplementary S5–S8 (publication bias tests)* |
| Certainty assessment | 15 | Describe any methods used to assess certainty (or confidence) in the body of evidence for an outcome. | Not reported |
| **RESULTS** | | |  |
| Study selection | 16a | Describe the results of the search and selection process, from the number of records identified in the search to the number of studies included in the review, ideally using a flow diagram. | *Figure 1 (PRISMA flow diagram)* |
|  | 16b | Cite studies that might appear to meet the inclusion criteria, but which were excluded, and explain why they were excluded. | *Section 2.1.2 Exclusion criteria + Results – Study selection* |
| Study characteristics | 17 | Cite each included study and present its characteristics. | *Results – Sample of studies + Supplementary S2 (Table list)* |
| Risk of bias in studies | 18 | Present assessments of risk of bias for each included study. | *Supplementary S4 (Quality assessment scores)* |
| Results of individual studies | 19 | For all outcomes, present, for each study: (a) summary statistics for each group (where appropriate) and (b) an effect estimate and its precision (e.g. confidence/credible interval), ideally using structured tables or plots. | *Tables 2–5 (results of individual studies)* |
| Results of syntheses | 20a | For each synthesis, briefly summarise the characteristics and risk of bias among contributing studies. | *Section 5.1 Summary of Overall Associations* |
|  | 20b | Present results of all statistical syntheses conducted. If meta-analysis was done, present for each the summary estimate and its precision (e.g. confidence/credible interval) and measures of statistical heterogeneity. If comparing groups, describe the direction of the effect. | *Sections 5.1–5.2 (meta-analysis results and heterogeneity indices)* |
|  | 20c | Present results of all investigations of possible causes of heterogeneity among study results. | *Section 5.3 (meta-regressions – sources of heterogeneity)* |
|  | 20d | Present results of all sensitivity analyses conducted to assess the robustness of the synthesized results. | *Supplementary S6–S8 (sensitivity analyses)* |
| Reporting biases | 21 | Present assessments of risk of bias due to missing results (arising from reporting biases) for each synthesis assessed. | *Section 5.1.2 and Supplementary S5 (publication bias)* |
| Certainty of evidence | 22 | Present assessments of certainty (or confidence) in the body of evidence for each outcome assessed. | Not Reported |
| **DISCUSSION** | | |  |
| Discussion | 23a | Provide a general interpretation of the results in the context of other evidence. | *Section 6.1 Main findings* |
|  | 23b | Discuss any limitations of the evidence included in the review. | *Section 6.4 Links with Clinical and Demographic Characteristics + limitations paragraph* |
|  | 23c | Discuss any limitations of the review processes used. | *End of Discussion – Methodological limitations* |
|  | 23d | Discuss implications of the results for practice, policy, and future research. | *Final paragraph of Discussion – Implications for future research* |
| **OTHER INFORMATION** | | |  |
| Registration and protocol | 24a | Provide registration information for the review, including register name and registration number, or state that the review was not registered. | *Section 2.5 Pre-registration and data availability (OSF link)* |
|  | 24b | Indicate where the review protocol can be accessed, or state that a protocol was not prepared. | *Section 2.5 – protocol accessible on OSF* |
|  | 24c | Describe and explain any amendments to information provided at registration or in the protocol. | Not applicable |
| Support | 25 | Describe sources of financial or non-financial support for the review, and the role of the funders or sponsors in the review. | *Funding statement (end of manuscript)* |
| Competing interests | 26 | Declare any competing interests of review authors. | *Competing interests statement (end of manuscript)* |
| Availability of data, code and other materials | 27 | Report which of the following are publicly available and where they can be found: template data collection forms; data extracted from included studies; data used for all analyses; analytic code; any other materials used in the review. | *Section 2.5 Pre-registration and data availability (OSF repository)* |

*From:* Page MJ, McKenzie JE, Bossuyt PM, Boutron I, Hoffmann TC, Mulrow CD, et al. The PRISMA 2020 statement: an updated guideline for reporting systematic reviews. BMJ 2021;372:n71. doi: 10.1136/bmj.n71. This work is licensed under CC BY 4.0. To view a copy of this license, visit <https://creativecommons.org/licenses/by/4.0/>
